# Supplementary material for: Factors associated with comprehensive knowledge of antenatal care and attitude towards its uptake among women delivered at home in rural Sehala Seyemit district, northern Ethiopia: A community-based cross-sectional study
Source: PLoS One. 2022 Oct 12;17(10):e0276125. doi: 10.1371/journal.pone.0276125 (PMC9555639; doi:10.1371/journal.pone.0276125)
Supplement: S1 File — (DOCX) [file pone.0276125.s001.docx]

**Details of Knowledge measurement**

The study participants were asked the following questions: 1) Starting early ANC is important 2) Pregnant women may have problems without ANC 3) ANC has to be recommended regardless of complications 4) Maternal waiting homes are important in area’s far from a health facility 5) Health facility delivery is safer and better than home delivery 6) Regular ANC medications can promote optimal growth of the unborn fetus 7) ANC can prevent complications 8) Alcohol drinking during pregnancy is bad for the fetus 9) Smoking during pregnancy is unsafe for the fetus 10) Do you know when to start ANC 11) At which stage of pregnancy fetal deformities most likely occur? 12) At what gestational age the first fetal movement is perceived by the woman? 13) The recommended number of ANC (in Ethiopian context) 14) what are the prevention methods of malaria during pregnancy? 15) What are the prevention of anemia during pregnancy? 16) What are the prevention of intestinal parasitic infection during pregnancy? 17) How tetanus can be prevented during pregnancy? 18) What are the danger signs during pregnancy or childbirth? 19) What are the components of birth preparedness and complication readiness plan? 20) What complications will a women face without using antenatal care or maternal health services? For questions 1-9, a score of 1 was given for “yes” and a score of 0 was given for “no” since the responses were yes or no. For question 10, a score of 1 was given for women who answered 3-4 months of pregnancy, otherwise 0. For question 11, a score of 1 was given for women who answered before the 3^rd^ month, otherwise 0. For question 12, a score of 1 was given for women who answered between the 4^th^ and 5^th^ months, otherwise 0. For question 13, a score of 1 was given for women who answered 4 and above, otherwise 0. For question 14, a score of 1 was given for women who answered through the use of insecticide-treated nets, antibiotics, voiding stagnant water, and close door and window, otherwise 0. For question 15, a score of 1 was given for women who answered avoid coffee, tea, and milk with a meal, use of routine supplemental iron, diet containing green and leafy vegetables, and diet containing red meat and liver, otherwise 0. For question 16, a score of 1 was given for women who answered use of mebendazole, avoid barefoot, and avoid eating raw meat, otherwise 0. For question 17, a score of 1 was given for women who answered tetanus toxoid vaccine and a score of 0 was given for women who said I don’t know. For question 18, a score of 1 was given for women who were able to list 3 danger signs, otherwise 0. For question 19, a score of 1 was given for women who were able to list 4 components from birth preparedness and complication readiness, otherwise 0. For question 20, a score of 1 was given for women who were able to list pregnancy and child birth related complications that could have a direct relation with the non-utilization of MHS, otherwise 0. Correct and/or “Yes” answers were coded as 1, whereas incorrect and/or “No”/don’t know answers were coded as 0.The minimum and maximum scores were 0 and 20, respectively. Thus, based on the summative score of variables designed to assess knowledge with a score above the mean was considered as knowledgeable (3,4,29).

**Supportive file for table 3 and description of each components of knowledge**

Table 3: Comprehensive knowledge of antenatal care among home-delivered women in rural Sehala Seyemit district, northern Ethiopia, 2020 (n = 653)

| **Variables** | **Frequency** | **Percentage (%)** |
| --- | --- | --- |
| Ever heard about ANC | Yes (556) | 85.1 |
|  | No (97) | 15.9 |
| Starting early ANC is important | Yes (367) | 56.2 |
|  | No (286) | 43.8 |
| Pregnant women may have problems without ANC | Yes (402) | 61.6 |
|  | No (251) | 38.4 |
| ANC has to be recommended regardless of complications | Yes (382) | 58.5 |
|  | No (271) | 41.5 |
| Maternal waiting homes are important in area’s far from a health facility | Yes (357) | 54.7 |
|  | No (296) | 45.3 |
| Health facility delivery is safer and better than home delivery | Yes (534) | 81.8 |
|  | No (119) | 18.2 |
| Regular ANC medications can promote optimal growth of the unborn fetus | Yes (426) | 65.2 |
|  | No (227) | 34.8 |
| ANC can prevent complications | Yes (452) | 69.2 |
|  | No (201) | 30.8 |
| Alcohol drinking during pregnancy is bad for the fetus | Yes (329) | 50.4 |
|  | No (324) | 49.6 |
| Smoking during pregnancy is unsafe for the fetus | Yes (585) | 96.9 |
|  | No (68) | 3.1 |
| Do you know when to start ANC | 3 to 4 months (362) | 55.4 |
|  | 4 to 5 months (90) | 13.8 |
|  | Before 3 months (23) | 3.5 |
|  | Don't know (178) | 27.3 |
| Perception of first fetal movement | In the 3^rd^ month (23)  Between the 4^th^ and 5^th^ months (271)  Don’t know (359) | 3.5  41.5  55 |
| At which stage of pregnancy fetal deformities most likely occur? | Below 3^rd^ month (124)  Between the 3^rd^ and the 7^th^ months (92)  After the 7^th^ month (12)  Don’t know (425) | 19  14.1  1.9  65 |
| The recommended number of ANC | Two (62)  Three (183)  Four and above (117)  Don’t know (291) | 9.5  28  17.9  44.6 |
| Prevention of malaria during pregnancy | Use of insecticide-treated nets (201)  Use of antibiotics (17)  Avoid stagnant water (28)  Close door and window (21)  Don't know (386) | 30.8  2.6  4.3  3.2  59.1 |
| Prevention of anemia during pregnancy | Avoid coffee, tea, and milk with a meal (29)  Use of routine iron (147)  Diet containing green and leafy vegetables (35)  Diet containing red meat and liver (24)  Don’t know (418) | 4.5  22.5  5.3  3.7  64 |
| Prevention of tetanus during pregnancy | Tetanus toxoid vaccine (194)  Don’t know (459) | 29.7  70.3 |
| Prevention of intestinal parasitic infection during pregnancy | Use of mebendazole (179)  Avoid barefoot (23)  Avoid eating raw meat (54)  Don’t know (397) | 27.4  3.5  8.3  60.8 |
| Knowledge of obstetric danger signs during pregnancy | Vaginal bleeding  Yes (402)  No (251)  Severe headache  Yes (220)  No (433)  Convulsion  Yes (176)  No (477)  Loss of consciousness  Yes (307)  No (346)  Epigastric pain  Yes (240)  No (413)  Pre-labor rupture of membrane  Yes (385)  No (268)  Facial or upper extremity edema  Yes (250)  No (403)  Decreased fetal movement  Yes (348)  No (305)  Blurring of vision  Yes (180)  No (473) | 61.6  38.4  33.7  66.3  26.9  73.1  47  53  36.8  63.2  58.9  41.1  38.3  61.7  53.3  46.7  27.5  72.5 |
| Knowledge of birth preparedness and complication readiness plan | Identify qualified birth attendants (12)  Identify health facility for delivery (23)  Arranged transport for emergency (86)  Saving money (121)  Prepare blood donor (4)  Prepare materials for delivery (105)  Identify danger signs and ready complication (19)  I don’t know (283) | 1.8  3.5  13.3  18.5  0.6  16.1  2.9  43.3 |
| What complications a woman will face without ANC? | Abortion (178)  Intrauterine fetal death (156)  Maternal complications (41)  Don’t know (278) | 27.3  23.9  6.3  42.5 |
| Overall comprehensive knowledge of ANC | Adequate knowledge (369)  Inadequate knowledge (284) | 56.5  43.5 |
