# Supplementary material for: Factors associated with comprehensive knowledge of antenatal care and attitude towards its uptake among women delivered at home in rural Sehala Seyemit district, northern Ethiopia: A community-based cross-sectional study
Source: PLoS One. 2022 Oct 12;17(10):e0276125. doi: 10.1371/journal.pone.0276125 (PMC9555639; doi:10.1371/journal.pone.0276125)
Supplement: S2 File — (DOCX) [file pone.0276125.s002.docx]

Annex1: English version of the questionnaire

Part I: Socio-demographic characteristics

| N.O | Questionnaire | Alternative choice for response | Skip code |
| --- | --- | --- | --- |
| 101 | How old are you? | (In years) |  |
| 102 | What is your educational level? | 1. Can’t read and write  2. Can read and write  3. Primary  4. Secondary  5. Diploma and above |  |
| 103 | What is your marital status? | 1. Single  2. Married  3. Divorced  4. Widowed  5. Separated |  |
| 104 | What is your occupation? | 1.House wife  2. Merchant  3. Government employee  4. self employed  5. Student  6. Others specify…………………………. |  |
| 105 | What is your religion? | 1. Orthodox Christian  2. Muslim  3. Protestant  4. Catholic  5. Other specify…………………………. |  |
| 107 | How many persons are living at home? |  |  |
| 108 | What is your husband educational level? | 1. Can’t read and write  2. Can read and write  3. Primary  4. Secondary  5. Diploma and above |  |
| 109 | What is your husband occupation? | 1. Daily labor 2. Farmer 3. Merchant 4. Government employee 5. Self employed 6. Student 7. Others specify…………………………. |  |
| 110 | Have you ever read newspapers? | 1. Yes 2. No | If no got to Q 112 |
| 111 | If yes, how often did you read? | 1. Every day 2. One times per week 3. Two times per week 4. Three and more |  |
| 112 | Have you ever heard radio? | 1. Yes 2. No | If no got to Q 114 |
| 113 | If yes, how often did you hear? | 1. Every day 2. One times per week 3. Two times per week 4. Three and more |  |
| 114 | How long does it take from your home to the nearby health facility on foot? |  |  |

Part II: Obstetric characteristics

| NO | Questioners | Alternative choices for response | Skip code |
| --- | --- | --- | --- |
| 201 | How many times did you give birth after 7 months? |  |  |
| 202 | Did you have ANC follow-up in your most recent pregnancy? | 1. Yes  2. No | If no got to 204 |
| 203 | How many ANC visits did you had on your most recent pregnancy? | 1. Once  2. Twice  3. Three times  4. Four and above |  |
| 204 | Who assisted your most recent delivery? | 1. 1. HEWs 2. 2. TBA 3. 3. Family |  |
| 205 | Did you have PNC visit in your most recent child? | 1. 1. Yes 2. 2. No | If no go to 207 |
| 206 | How many PNC visits did you have? | 1. 1. One 2. 2. Two 3. 3. Three or more |  |
| 207 | Did health extension workers visited you after your recent pregnancy? | 1. 1. Yes 2. 2. No |  |
| 208 | Did you have a history of neonatal death? | 1. 1. Yes   2. No |  |
| 209 | Do you have history of obstetric danger signs | 1. 1. Yes 2. 2. No |  |
| 210 | Husband/Partner involvement related questions on MNCH services | | |
| I | Did your husband go with you for ANC follow up at least once in your most recent pregnancy? | 1. Yes  2. No |  |
| II | Did your husband provide transport/gave money for transport during your recent pregnancy or delivery? | 1.Yes  2. No |  |
| III | Did your husband accompany to the hospital during labor for your recent delivery? | 1. Yes  2. No |  |
| IV | Did your husband discuss with health care providers during your recent pregnancy or delivery? | 1. Yes  2. No |  |
| V | Did your husband look after the child at home/stay with babies while you are outside home? | 1. Yes  2. No |  |
| VI | Did your husband bath newborn/infant while you are busy? | 1. Yes  2. No |  |
| VII | Did your husband buy clothes/other things for infants/neonates? | 1. Yes  2. No |  |
| VIII | Did your husband go with you for immunization services? | 1. Yes  2. No |  |
| IX | Did your husband assisted you while you breastfeed the newborn? | 1. Yes  2. No |  |
| 212 | Is the pregnancy wanted, planned and supported? | 1. 1. Yes 2. 2. No |  |
| 213 | Are you intended to deliver in a health facility for the next pregnancy? | 1. 1. Yes 2. 2. No |  |
| 214 | Why you are not using antenatal care and institutional delivery? |  |  |

Part III: Mothers knowledge related questions

| No | Questionnaire | Alternative choice for response | Skip code |
| --- | --- | --- | --- |
| 301 | Have you ever heard about ANC? | 1. 1.Yes 2. 2. No |  |
| 302 | Do you know when to start the first ANC visit? | 1. 1. Before 3 months 2. 2. 3 to 4 months 3. 3. 4 to 5 months |  |
| 303 | Do you think ANC can prevent complications? | 1. 1. Yes 2. 2. No |  |
| 304 | Pregnant women may have problems without ANC? | 1. 1. Yes 2. 2. No |  |
| 305 | Regular ANC medications can promote optimal growth of unborn child? | 1.Yes  2. No |  |
| 306 | Health facility delivery is safer and better than home delivery? | 1.Yes   1. 2. No |  |
| 307 | Do you think starting ANC early will be important? | 1.Yes   1. 2. No |  |
| 308 | Do you know the recommended frequency of ANC visits? | - - - 1. One       2. Two       3. Three       4. 4 and above |  |
| 309 | Do you think ANC has to be recommended regardless of complications? | 1.Yes  2. No |  |
| 310 | At which stage of pregnancy fetal deformity most likely occur? | Below 3^rd^ moth  Between the 3^rd^ and the 7^th^ month  After the 7^th^ month  Don’t know |  |
| 311 | Perception of first fetal movement | At the 3^rd^ month  Between the 4^th^ and 5^th^ month  Don’t know |  |
| 312 | Do you know how to prevent malaria during pregnancy? | 1.Yes  2. No |  |
| 313 | If yes for Q 312, how do you prevent malaria during pregnancy? |  |  |
| 314 | Do you know how to prevent iron deficiency anemia during pregnancy? | 1.Yes  2. No |  |
| 315 | If yes for Q314, how do you prevent iron deficiency anemia during pregnancy? |  |  |
| 316 | Do you know how to prevent intestinal parasites during pregnancy? | 1.Yes  2. No |  |
| 317 | If yes for Q316, how do you prevent intestinal parasites during pregnancy? |  |  |
| 318 | When we say a women have optimum ANC? | 1. 4 and above 2. Less than 4 |  |
| 319 | Do you think maternal waiting homes are important for a pregnant women? | 1.Yes  2. No |  |
| 320 | Have you ever used maternal waiting homes in your recent pregnancy? | 1.Yes  2. No |  |
| 321 | Do you know the obstetric danger signs during pregnancy? | 1.Yes  2. No |  |
| 322 | If yes for Q321, list all the obstetric danger signs during pregnancy | 1. Vaginal bleeding 2. Blurring of vision 3. Epigastric pain 4. High grade fever 5. Convulsion 6. Loss of consciousness 7. Decreased/absent fetal movement 8. Others |  |
| 323 | Do you know smoking cigarate and drinking alcohol is unsafe for the fetus? | 1. Yes 2. No |  |
| 324 | Do you how to prevent malaria during pregnancy | 1. Use of ITN 2. Eliminating stagnant water 3. Use of antimalarial prophylaxis 4. Other specify |  |
| 325 | Do you know how to prevent anemia during pregnancy? | 1. Use of iron as recommended 2. Avoid milk, coffee and tea with meals 3. Eating green leafy vegetables 4. Don’t know |  |
| 326 | Do you know how to prevent helminthic infection during pregnancy? | 1. Use of mebendazole 2. Avoid eating raw meat 3. Avoid bare foot 4. Don’t know |  |
| 327 | Do you how to prevent tetanus during pregnancy? | 1. Use of TT vaccine 2. Don’t know |  |
| 328 | What complications a woman will face without ANC? |  |  |
| Part Iv: Attitude related questions | | | |
| 401 | Do you want to have ANC follow-up next time? | Strongly agree  Agree  Neutral  Disagree  Strongly disagree |  |
| 402 | Do you agree that healthcare professionals providing prenatal care is good? | Strongly agree  Agree  Neutral  Disagree  Strongly disagree |  |
| 403 | Do you agree that all pregnant mothers should have ANC follow-up? | Strongly agree  Agree  Neutral  Disagree  Strongly disagree |  |
| 404 | Do you agree that timely ANC follow-up will be safer for both mother and baby during labor and delivery? | Strongly agree  Agree  Neutral  Disagree  Strongly disagree |  |
| 405 | Do you agree that husbands should be present during ANC follow-up? | Strongly agree  Agree  Neutral  Disagree  Strongly disagree |  |
| 406 | Do you want to pay for ANC if it is with fee? | Strongly agree  Agree  Neutral  Disagree  Strongly disagree |  |
| 407 | Advice regarding proper health during pregnancy can be gotten outside the hospital? | Strongly agree  Agree  Neutral  Disagree  Strongly disagree |  |
| 408 | Follow up during pregnancy may decrease antenatal and postnatal complications | Strongly agree  Agree  Neutral  Disagree  Strongly disagree |  |
| 409 | Husbands should be present during ANC follow-up | Strongly agree  Agree  Neutral  Disagree  Strongly disagree |  |

Part V: Decision making related questions

| No | Questionnaire | Alternative choice for response | Skip code |
| --- | --- | --- | --- |
| 501 | Who decisions about health care for yourself? | 1. Me alone 2. My husband alone 3. Both of us 4. Others |  |
| 502 | Who decides on large household purchase or sell? | 1. Me alone 2. My husband alone 3. Both of us 4. Others |  |
| 503 | Who decides on intrahousehold resource allocation/ daily household purchases? | 1. Me alone 2. My husband alone 3. Both of us 4. Others |  |
| 504 | Who decides on where and when to seek medical care for sick newborns/children? | 1. Me alone 2. My husband alone 3. Both of us 4. Others |  |
| 505 | Who decides on visits of family, friends or relatives? | 1. Me alone 2. My husband alone 3. Both of us 4. Others |  |
| 506 | Who decides when to have an additional child? | 1. Me alone 2. My husband alone 3. Both of us 4. Others |  |
| 507 | Who usually decides how your partner’s/husband earnings will be used? | 1. Me alone 2. My husband alone 3. Both of us 4. Others |  |
| 508 | Who decides to go for ANC visit, PNC visit, where to deliver and infant immunization? | 1. Me alone 2. My husband alone 3. Both of us 4. Others |  |
| 509 | Who usually decides what foods to be cooked each day? | 1. Me alone 2. My husband alone 3. Both of us 4. Others |  |
