# Supplementary material for: Factors associated with comprehensive knowledge of antenatal care and attitude towards its uptake among women delivered at home in rural Sehala Seyemit district, northern Ethiopia: A community-based cross-sectional study
Source: PLoS One. 2022 Oct 12;17(10):e0276125. doi: 10.1371/journal.pone.0276125 (PMC9555639; doi:10.1371/journal.pone.0276125)
Supplement: Attachment — Submitted filename: TM_PONE-D-21-01426_reviewer.pdf [file pone.0276125.s003.pdf]

PLOS ONE

Comprehensive Knowledge of Antenatal Care, Attitude towards Its Uptake and Associated Factors among Home-Delivered Women in Rural Sehala Seyemit District, Northern Ethiopia: A Community-Based Cross-Sectional Study

--Manuscript Draft--

**Manuscript Number:**	PONE-D-21-01426
**Article Type:**	Research Article
**Full Title:**	Comprehensive Knowledge of Antenatal Care, Attitude towards Its Uptake and Associated Factors among Home-Delivered Women in Rural Sehala Seyemit District, Northern Ethiopia: A Community-Based Cross-Sectional Study
**Short Title:**	Knowledge of Antenatal Care, Attitude towards Its Uptake and Associated Factors among Home-Delivered Women
**Corresponding Author:**	Azmeraw Ambachew Kebede, MSc<br>University of Gondar College of Medicine and Health Sciences<br>Gondar, Amhara regional State ETHIOPIA
**Keywords:**	ANC; Attitude; Ethiopia; Home-delivery; Knowledge
**Abstract:**	Introduction<br><br>Despite an endeavor made to drop maternal and neonatal mortality, Ethiopia remains amongst the highest mortality rate globally. Therefore, this study aimed to assess comprehensive knowledge of antenatal care, attitude towards its uptake, and associated factors among home-delivered women in rural Sehala Seyemit district, northern Ethiopia.<br><br>Methods<br><br>A community-based cross-sectional study was conducted from September 1st to October 15th, 2020. A multi-stage sampling technique was used to select 653 women. The data was collected using a semi-structured and pretested questionnaire via face to face interview. Data were entered into Epi info 7.1.2 and analyzed by SPSS version 25. Both bivariable and multivariable logistic regression analyses were undertaken to identify factors associated with women's knowledge of antenatal care and attitude towards its uptake. Statistically significant association of variables was determined based on adjusted odds ratio with a 95% confidence interval and p-value of ≤0.05.<br><br>Results<br><br>Women's knowledge of antenatal care and positive attitude towards its uptake was 56.5% (95% CI; 52.6, 60.6) and 75.2% (95% CI; 71.8, 78.5) respectively. Older age (AOR= 7.2; 95% CI: 3.43, 15.1), media exposure (AOR= 3.69; 95% CI: 2.41, 5.65), history of abortion (AOR= 11.6; 95% CI: 3.3, 14.6), time to reach health facility (AOR= 4.58; 95% CI: 3.05, 6.88) and history of obstetric danger signs (AOR= 7.3; 95% CI: 3.92, 13.64) were factors significantly associated with knowledge of antenatal care. Furthermore, decision-making power (AOR= 8.3; 95% CI: 4.8, 13.83), knowledge of antenatal care (AOR= 2.2; 95% CI: 1.26, 3.71), delivery by health extension workers (AOR= 2.3; 95% CI: 1.1, 5.1), and media exposure (AOR= 2.27; 95% CI: 1.30, 3.97) were predictors of a favorable attitude towards antenatal care.<br><br>Conclusion<br><br>Although the majority of women in the present study had a favorable attitude towards antenatal care uptake, their knowledge level was inadequate. Strengthening access to transportation, mass media, involvement in household decision-making, and encouraging women to deliver at a health facility by a skilled provider will increase women's knowledge and attitude regarding antenatal care that successively increases healthcare access.
**Order of Authors:**	Azmeraw Ambachew Kebede, MSc

Powered by Editorial Manager® and ProduXion Manager® from Aries Systems Corporation

	Birhan Tsegaw Taye
	Kindu Yinges Wondie
**Additional Information:**	
**Question**	**Response**
**Financial Disclosure**<br><br>Enter a financial disclosure statement that describes the sources of funding for the work included in this submission. Review the submission guidelines for detailed requirements. View published research articles from *PLOS ONE* for specific examples.<br><br>This statement is required for submission and **will appear in the published article** if the submission is accepted. Please make sure it is accurate.<br><br>**Unfunded studies**<br>Enter: *The author(s) received no specific funding for this work.*<br><br>**Funded studies**<br>Enter a statement with the following details:<br>• Initials of the authors who received each award<br>• Grant numbers awarded to each author<br>• The full name of each funder<br>• URL of each funder website<br>• Did the sponsors or funders play any role in the study design, data collection and analysis, decision to publish, or preparation of the manuscript?<br>• **NO** - Include this sentence at the end of your statement: *The funders had no role in study design, data collection and analysis, decision to publish, or preparation of the manuscript.*<br>• **YES** - Specify the role(s) played.<br><br>* typeset	The authors received no specific funding for this work.
**Competing Interests**<br><br>Use the instructions below to enter a competing interest statement for this submission. On behalf of all authors, disclose any competing interests that could be perceived to bias this work—acknowledging all financial support and any other relevant financial or non-	The authors have declared that no competing interests exist

financial competing interests.<br><br>This statement is required for submission and **will appear in the published article** if the submission is accepted. Please make sure it is accurate and that any funding sources listed in your Funding Information later in the submission form are also declared in your Financial Disclosure statement.<br><br>View published research articles from *PLOS ONE* for specific examples.<br><br>**NO authors have competing interests**<br><br>Enter: *The authors have declared that no competing interests exist.*<br><br>**Authors with competing interests**<br><br>Enter competing interest details beginning with this statement:<br><br>*I have read the journal's policy and the authors of this manuscript have the following competing interests: [insert competing interests here]*<br><br>* typeset	
**Ethics Statement**<br><br>Enter an ethics statement for this submission. This statement is required if the study involved:<br><br>• Human participants<br>• Human specimens or tissue<br>• Vertebrate animals or cephalopods<br>• Vertebrate embryos or tissues<br>• Field research<br><br>Write "N/A" if the submission does not require an ethics statement.<br><br>General guidance is provided below.<br>Consult the submission guidelines for	We conducted the study under the declaration of Helsinki. Ethical approval was obtained from the Institutional Ethical Review Board of Debre Berhan University (protocol number; P005/20). A formal letter of administrative support was gained from the Sehala Seyemit Woreda health office. Both oral and written informed consent was collected from each of the study members was obtained after a clear explanation of the aim of the study.

detailed instructions. **Make sure that all information entered here is included in the Methods section of the manuscript.**<br><br>**Format for specific study types**<br><br>**Human Subject Research (involving human participants and/or tissue)**<br>• Give the name of the institutional review board or ethics committee that approved the study<br>• Include the approval number and/or a statement indicating approval of this research<br>• Indicate the form of consent obtained (written/oral) or the reason that consent was not obtained (e.g. the data were analyzed anonymously)<br><br>**Animal Research (involving vertebrate animals, embryos or tissues)**<br>• Provide the name of the Institutional Animal Care and Use Committee (IACUC) or other relevant ethics board that reviewed the study protocol, and indicate whether they approved this research or granted a formal waiver of ethical approval<br>• Include an approval number if one was obtained<br>• If the study involved *non-human primates*, add *additional details* about animal welfare and steps taken to ameliorate suffering<br>• If anesthesia, euthanasia, or any kind of animal sacrifice is part of the study, include briefly which substances and/or methods were applied<br><br>**Field Research**<br><br>Include the following details if this study involves the collection of plant, animal, or other materials from a natural setting:<br>• Field permit number<br>• Name of the institution or relevant body that granted permission	
**Data Availability**<br><br>Authors are required to make all data underlying the findings described fully available, without restriction, and from the time of publication. PLOS allows rare	Yes - all data are fully available without restriction

exceptions to address legal and ethical concerns. See the PLOS Data Policy and FAQ for detailed information.<br><br>A Data Availability Statement describing where the data can be found is required at submission. Your answers to this question constitute the Data Availability Statement and **will be published in the article**, if accepted.<br><br>**Important:** Stating 'data available on request from the author' is not sufficient. If your data are only available upon request, select 'No' for the first question and explain your exceptional situation in the text box.<br><br>Do the authors confirm that all data underlying the findings described in their manuscript are fully available without restriction?	
**Describe where the data may be found in full sentences. If you are copying our sample text, replace any instances of XXX with the appropriate details.**<br><br>• If the data are **held or will be held in a public repository**, include URLs, accession numbers or DOIs. If this information will only be available after acceptance, indicate this by ticking the box below. For example: *All XXX files are available from the XXX database (accession number(s) XXX, XXX.*).<br>• If the data are all contained **within the manuscript and/or Supporting Information files**, enter the following: *All relevant data are within the manuscript and its Supporting Information files.*<br>• If neither of these applies but you are able to provide **details of access elsewhere**, with or without limitations, please do so. For example:<br><br>*Data cannot be shared publicly because of [XXX]. Data are available from the XXX Institutional Data Access / Ethics Committee (contact via XXX) for researchers who meet the criteria for*	All the data is available on the corresponding author based on request.

Powered by Editorial Manager® and ProduXion Manager® from Aries Systems Corporation

access to confidential data.<br><br>*The data underlying the results presented in the study are available from (include the name of the third party and contact information or URL).*<br>• This text is appropriate if the data are owned by a third party and authors do not have permission to share the data.<br><br>* typeset	
Additional data availability information:	

Powered by Editorial Manager® and ProduXion Manager® from Aries Systems Corporation

Manuscript

Click here to access/download;Manuscript;ANC knowledge manuscript.docx

Type of manuscript:

Original research article

Title:

Comprehensive Knowledge of Antenatal Care, Attitude towards Its Uptake and Associated Factors among Home-Delivered Women in Rural Sehala Seyemit District, Northern Ethiopia: A Community-Based Cross-Sectional Study

Name of Authors:

Azmeraw Ambachew Kebede [1], Birhan Tsegaw Taye [2], Kindu Yinges Wondie [1]

Affiliation:

[1] Department of Clinical Midwifery, School of Midwifery, College of Medicine and Health Sciences, University of Gondar, Gondar, Ethiopia; [2] Department of Midwifery, College of Medicine and Health Sciences, Debre Berhan University, Debre Berhan, Ethiopia

Correspondence: Azmeraw Ambachew Kebede

Po. Box 196 Gondar, Ethiopia

Tel: +251910880232

Email: azmuzwagholic@gmail.com

Abstract

Introduction: Despite an endeavor made to drop maternal and neonatal mortality, Ethiopia remains amongst the highest mortality rate globally. Therefore, this study aimed to assess comprehensive knowledge of antenatal care, attitude towards its uptake, and associated factors among home-delivered women in rural Sehala Seyemit district, northern Ethiopia.

Methods: A community-based cross-sectional study was conducted from September 1st to October 15th, 2020. A multi-stage sampling technique was used to select 653 women. The data was collected using a semi-structured and pretested questionnaire via face to face interview. Data were entered into Epi info 7.1.2 and analyzed by SPSS version 25. Both bivariable and multivariable logistic regression analyses were undertaken to identify factors associated with women's knowledge of antenatal care and attitude towards its uptake. Statistically significant association of variables was determined based on adjusted odds ratio with a 95% confidence interval and p-value of ≤0.05.

Results: Women's knowledge of antenatal care and positive attitude towards its uptake was 56.5% (95% CI; 52.6, 60.6) and 75.2% (95% CI; 71.8, 78.5) respectively. Older age (AOR= 7.2; 95% CI: 3.43, 15.1), media exposure (AOR= 3.69; 95% CI: 2.41, 5.65), history of abortion (AOR= 11.6; 95% CI: 3.3, 14.6), time to reach health facility (AOR= 4.58; 95% CI: 3.05, 6.88) and history of obstetric danger signs (AOR= 7.3; 95% CI: 3.92, 13.64) were factors significantly associated with knowledge of antenatal care. Furthermore, decision-making power (AOR= 8.3; 95% CI: 4.8, 13.83), knowledge of antenatal care (AOR= 2.2; 95% CI: 1.26, 3.71), delivery by health extension workers (AOR= 2.3; 95% CI: 1.1, 5.1), and media exposure (AOR= 2.27; 95% CI: 1.30, 3.97) were predictors of a favorable attitude towards antenatal care.

Conclusion: Although the majority of women in the present study had a favorable attitude towards antenatal care uptake, their knowledge level was inadequate. Strengthening access to transportation, mass media, involvement in household decision-making, and encouraging women to deliver at a health facility by a skilled provider will increase women's knowledge and attitude regarding antenatal care that successively increases healthcare access.

Keywords: ANC, Attitude, Ethiopia, Home-delivery, Knowledge

Introduction

Antenatal care (ANC) is the care provided to all pregnant women by qualified health professionals during pregnancy (1). It is given to all pregnant women regardless of perquisites. However, the number of antenatal contacts might increase in women with medical problems. Antenatal care aids pregnant women and their families to become arranged and prepared for the consequences related to pregnancy and childbirth (2). Accordingly, the primary purpose of ANC is to ascertain pregnant women who are at risk, support them to be healthy, and prevent disease and improve healthcare access throughout the maternal continuity of care (3).

Antenatal care gives great opportunities to keep pregnant women healthy and decreases the hazards of pregnancy adverse events and neonatal mortality, and morbidity (4). Besides, evidences support that ANC enhances women's knowledge of maternal and neonatal danger signs (5,6), a roadmap for health facility delivery (7), and increases postnatal care utilization (8).

Globally, about 295,000 women died due to complications related to pregnancy and childbirth in the year 2017 (9). Seven out of ten women died from Sub-Saharan Africa (9). This high rate of maternal mortality due to poor use of health services. Once more, inadequate knowledge of women about maternal health service utilization could be listed as a prior reason for the low utilization of health services. As a result, building up women's knowledge of ANC and attitudes towards its uptake can drop the tragic maternal mortality and morbidity. Because sufficient knowledge of ANC can increase the uptake of maternal health services (optimum ANC, health facility delivery, and postnatal care utilization). Therefore, the care provided during pregnancy is a precondition for the eventual reduction of maternal, neonatal, and perinatal mortality and morbidities associated with pregnancy and childbirth (10). Besides, raising the awareness of mothers about antenatal care and strengthening their knowledge will help maintain good maternal and child health. Evidence indicated that lack of awareness about maternal health services hampers maternal health care service utilization (11).

In 2016, the World Health Organization (WHO) recommends that all mothers should have at least eight antenatal care to ensure better maternal and neonatal health (10). However, Ethiopia still uses the four ANC visit recommendations. Despite great emphasis given to

maternal and neonatal health, ensuring the optimal health of both the mother and newborns is the foremost challenge in Sub-Saharan Africa (12).

According to the 2016 Ethiopian demographic health survey report, the maternal mortality ratio was 412 per 100, 00 live births (13) and the neonatal mortality rate was 30 per 1000 live births (14). Similarly, the 2019 Ethiopian mini demographic health survey concluded that 57% of women did not receive optimum ANC in their recent pregnancy. Moreover, 52% of women gave birth at home (14).

Many studies have determined the utilization and factors affecting ANC in Ethiopia (2,15–22). However, a few studies were conducted on women's knowledge of ANC and attitudes towards its uptake (3,23). Adequate knowledge of ANC increases pregnant women's adherence to supplemental drugs (for example, folic acid, vitamins, and de-worming) and able to identify danger signs of pregnancy, and taking an action promptly (24). Besides, the provision of quality maternal healthcare services helps to prevent and manage pregnancy-related maternal and neonatal complications and deaths (25–27).

Studies elsewhere described that women's knowledge of antenatal care was 79.2% (4), 92.3%(24), and 85.3% (28). Evidences in Ethiopia also determined that knowledge of antenatal care was 88.2% (3) and 90.7% (23). Besides, findings showed that the attitude of women towards antenatal care uptake was 90.3% in Saudi (24), 96.0% in Libya (28), and 69.6% in Mizan, Ethiopia (3). However, most of these studies failed to apply logistic regression analysis to establish factors correlated with women's knowledge of ANC and attitude towards its uptake. Also, there are limited studies conducted in Ethiopia that address home-delivered women on the aforementioned topic.

Increasing women's knowledge of antenatal care and attitude towards its uptake is the pillar to expand maternity and child healthcare utilization throughout the maternal continuum of care. Therefore, this study aimed to determine comprehensive knowledge of antenatal care, attitude towards its uptake, and associated factors among home-delivered women in rural Sehala Seyemit district, Northern Ethiopia.

Methods and Materials

Study Design, Setting, and Period

A Community-based cross-sectional study was conducted from September 1st to October 15th, 2020. This study was conducted in rural Sehala Seyemit district, Waghimra zone, Amhara regional state, Northern Ethiopia. Sehala Seyemit district is located 285 km northeast of Bahir Dar (the capital city of Amhara regional state) and about 799 km north of Addis Ababa (the capital city of Ethiopia). Accessing health services in the district is difficult because of the lack of transportation to each *“kebeles”* (which is the smallest administrative unit in Ethiopia). The district has 13 *“kebeles”*; 12 rural and 1 urban “kebeles”. Currently, the district has a population of 39,435. Over 90% of the population are farmers. Moreover, there are 3 health centers and 13 health posts serving the community (Sehala Seyemit Woreda report, unpublished data).

Study population

All home-delivered women in the last two years in the selected *“kebeles”* during the data collection period were the study population. All critically ill women throughout the data collection period were excluded.

Sample Size Determination and Sampling Procedure

The sample size for this study was determined by using a single population proportion formula by considering the following assumptions: women’s attitude towards ANC 70.6% (3), 95% level of confidence, and 5% margin of error.

$$n = \frac{Z\alpha/2)^2 p(1-p)}{d^2} = n = \frac{(1.96)^{2}{*}0.706(1-0.706)}{(0.05)2} = 319$$

Where, n= required sample size, α = level of significant, z= standard normal distribution curve value for 95% confidence level= 1.96, p= women’s attitude towards ANC, d= margin of error. By considering a design effect of 2 (since multistage sampling) and a 5% non-response rate, the minimum adequate sample size was 670. A multistage sampling technique was employed to select the study participants. In the first stage, eight kebeles were selected randomly among the 12 rural “*kebeles*”. The lists of home-delivered women were obtained from health extension workers (HEWs) and local administrators. The sampling frame was designed by numbering the list of women. Then, the researchers distributed the total sample size to each selected *“kebeles”* proportionally. Finally, the

women were selected by simple random sampling technique using a table of random generation.

Variables of the Study

Outcome Variables

Women's knowledge of antenatal care (Adequate/ Inadequate)

Women's attitude towards antenatal care uptake (Favorable/ Unfavorable)

Independent Variables

Age of the women, marital status, women's educational status, women's occupation, husband educational status, husband occupation, family size, exposure to mass media, time to reach the nearby health facility, parity, history of ANC, number of ANC, birth assistant, husband involvement in maternal and children's health, household decision-making power, history of abortion, history of neonatal death, history of obstetric danger signs during pregnancy and status of the pregnancy.

Measurements

Comprehensive Knowledge of ANC: Includes knowledge of antenatal care and pregnancy, knowledge of obstetric danger signs, knowledge of birth preparedness and complication readiness, knowledge of malaria prevention, knowledge of anemia prevention, knowledge of helminthic infection prevention, and knowledge of tetanus prevention during pregnancy. A total of 21 open and close-ended questions were designed to assess the comprehensive knowledge of antenatal care. Thus, based on the summative score of variables designed to assess knowledge with a score above 60the mean was considered as knowledgeable (3,4,29).

Women's Attitude: Women's attitude towards antenatal care was measured using 9 questions: 1) Want to have ANC follow up for next time 2) Intention to deliver in a health facility for the next pregnancy 3) Health care professionals providing antenatal care is good 4) All pregnant women should have ANC follow up 5) Timely ANC follow up will be safer for both mother and baby 6) Want to pay for ANC if it is with fee 7) Husbands should be present during ANC follow-up 8) Advice regarding proper health during pregnancy can be gotten outside the hospital 9) Follow up during pregnancy may decrease antenatal and postnatal complications**.** Each question has a five-point Likert scale (1= strongly disagree,

2= disagree, 3 = neutral, 4= agree, 5= strongly agree). The total score was 9–45 and women who scored above the mean value were considered as having a favorable attitude (3,29).

Husband Involvement: Husband involvement in maternal and child health-related activities was measured using 9 questions: 1) did your husband go with you for ANC follow up at least once in your most recent pregnancy? 2) Did your husband provide transport/gave money for transport during your recent pregnancy or delivery? 3) Did your husband accompany you to the hospital during labor for your recent delivery? 4) Did your husband discuss with health care providers during your recent pregnancy or delivery? 5) Did your husband look after the child at home/stay with the babies while you are outside home? 6) Did your husband bathe a newborn/infant while you are busy? 7) Did your husband buy clothes/other things for infants/neonates? 8) Did your husband go with you for immunization services? 9) Did your husband assisted you while you breastfeed the newborn/infant? Each question was coded as 0 for "no" and 1 for "yes". The total score ranged from 0-9 and a score of above the mean was considered as husband involved Based on the summative score of variables designed to assess husband involvement with a score above the mean was considered as involved (30,31)

Household Decision-Making Power: Women's decision-making power was assessed using 9 questions: 1) who decides about health care for you? 2) Who decides on the large household purchase or sell? 3) Who decides on intrahousehold resource allocation/ daily household purchases? 4) Who decides on where and when to seek medical care for sick newborns/children? 5) Who decides on visits of family, friends, or relatives? 6) Who decides when to have an additional child? 7) Who usually decides how your partner's/husband earnings will be used? 8) Who decides to go for ANC visit, postnatal (PNC) visit, where to deliver, and infant immunization? 9) Who usually decides what foods to be cooked each day? The possible answers were me alone which was coded as 2, both of us which was coded as 1, the husband alone which was coded as 0. The score was ranged from 0 to 18 and a woman who scored above the mean was considered as having higher household decision-making power (32).

Data Collection Instruments, Procedures, and Quality Control

The data collection tool was developed by reviewing literature (3,4,23) and collected using a semi-structured interviewer-administered questionnaire through face-to-face interviews. Initially, the questionnaire was prepared in English and translated to Amharic language and back to English to ensure consistency. The questionnaire contains socio-demographic

characteristics, reproductive and maternity healthcare characteristics, husband involvement in maternal and child health-related activities, household decision-making power, and questions assessing women's comprehensive knowledge of antenatal care, and attitude towards its uptake. The questionnaire was assessed by a group of researchers (three in the field of maternal and child health, one in the field of public health, and two midwives in the hospital) to evaluate and enhance the items in the question. Before the actual data collection, we did a pretest on 34 women at Ziquala Woreda which has similar socio-cultural and living standards as the study population. Eight female HEWs and four male Diploma in midwifery holders were recruited for data collection and supervision, respectively. Two-days of training were given regarding the overall data collection process. During the data collection, the questionnaire was checked for completeness daily by the supervisors.

Data Processing and Analysis

Data were checked, coded, and entered into EPI INFO version 7.1.2, and were exported to SPSS version 25 for analysis. Before analysis re-coding, transforming, computing, and categorizing of variables were done. Descriptive statistics were used to show participants' characteristics, comprehensive knowledge of antenatal care, and attitude towards its uptake. Binary logistic regression analysis was fitted to identify statistically significant independent variables, and variables having a p-value of less than 0.2 were included in the multivariable logistic regression for controlling confounders. In multivariable logistic regression, a p-value of <0.05 with a 95% CI for odds ratio was employed to ascertain the significant association.

Ethics Approval and Consent to Participate

We conducted the study under the declaration of Helsinki. Ethical approval was obtained from the Institutional Ethical Review Board of Debre Berhan University (protocol number; P005/20). A formal letter of administrative support was gained from the Sehala Seyemit Woreda health office. Both oral and written informed consent was collected from each of the study members was obtained after a clear explanation of the aim of the study.

Result

Socio-Demographic Characteristics of Study Participant

In this study, a total of 670 women were interviewed. Seventeen women were excluded for their incomplete data, giving a 97.5% response rate. The mean age of the respondents was 26.4 years (SD ±4.93) and more than two-thirds of the participant's age was between the age group of 21-30 years. Most (97.2%) of the participants were in marital union and 384 (58.8%) of them were unable to read and write by education. Over four-fifths of the women were farmers by occupation. Concerning the occupation of the husband, 85% were farmers and slightly more than half (51%) of them were unable to read and write **(Table1).**

Reproductive History and Maternity Healthcare Service-Related Characteristics

Of the total study members, only 198 (30.3%) of women had at least one ANC visit in their recent pregnancy. About 58% of participants were assisted by HEWs for their recent delivery. More than half (56.6%) of women got the husband's involvement in maternity and childcare-related activities **(Table 2).**

Women's Comprehensive Knowledge of Antenatal Care

Overall, 56.5% of women had adequate knowledge of antenatal care (95 % CI; 52.6, 60.6). The majority (85.1%) of respondents ever heard about antenatal care. More than half (56.2%) of women knew the importance of early ANC visits. Only 17.9% of women had known the recommended number of antenatal care **(Table 3).**

Participant's Attitude towards Antenatal Care Uptake

Two-thirds of women had a favorable attitude towards antenatal care uptake (95% CI; 71.8, 78.5). About 148 (22.7%) women strongly agreed that husbands should present during ANC visits. Nearly 12% of women had a desire to pay for ANC if it is with a cost **(Table 4).**

Barriers to Use Antenatal Care and Institutional Delivery

All women who were part of this study gave their recent birth at home. Besides, the majority of women didn't have ANC visits for their recent pregnancy. The main reasons mentioned by the participants for not having ANC and institutional delivery were long travel time and/or lack of transportation, considering ANC may not be necessary and having a workload **(Figure 1).**

Factors Associated with Women's Knowledge of Antenatal Care

Multivariable logistic regression analysis revealed that women's age, media exposure, history of abortion, had experienced obstetric danger signs in the recent pregnancy, and time to reach the health facility were factors significantly associated with women's comprehensive knowledge of antenatal care.

Women who had mass media exposure were 3.69 times more likely to have had adequate knowledge of ANC compared to women who didn't have media exposure (AOR= 3.69; 95% CI: 2.41, 5.65).

Women who have had a history of abortion were 11.6 times more likely to have had adequate knowledge of antenatal care compared to their counterparts (AOR= 11.6; 95% CI: 3.3, 14.6).

Similarly, the odds of having adequate knowledge of antenatal care among women who travel less than one hour to reach the health facility were four (AOR= 4.58; 95% CI: 3.05, 6.88) times compared to women who travel for more than one hour.

This study also found that women greater than 30 and 20-30 years old were seven (AOR= 7.2; 95% CI: 3.43, 15.1) and three (AOR= 3.26; 95% CI: 1.73, 6.15) times more likely to have had adequate knowledge of antenatal care when compared to women less than or equal to 20 years old respectively.

Moreover, those women who have had a history of obstetric danger signs during their recent pregnancy were seven times (AOR= 7.3; 95% CI: 3.92, 13.64) more likely to have had adequate knowledge of antenatal care compared to those women who haven't had danger signs **(Table 5).**

Factors Associated with Women's Attitude towards Antenatal Care Uptake

On the multivariable logistic regression analysis knowledge of antenatal care, household decision-making power, delivery assisted by health extension workers, and exposure to mass media were factors statically significant with a favorable attitude of women towards antenatal care uptake.

The odds of having a favorable attitude towards antenatal care uptake among women who had adequate knowledge of antenatal care were two times (AOR= 2.2; 95% CI: 1.26, 3.71) compared to women who had poor knowledge of antenatal care.

The present study revealed that women who had higher household decision-making power were 8 times (AOR= 8.3; 95% CI: 4.8, 13.83) more likely to have had a favorable attitude towards antenatal care uptake compared to women who had less decision-making power.

Similarly, the likelihood of having a favorable attitude towards antenatal care uptake among women who were assisted by health extension workers for their recent delivery was two times (AOR= 2.3; 95% CI: 1.1, 5.1) compared to women who were assisted by family members.

Lastly, those women who were exposed to mass media were 2.27 (AOR= 2.27; 95% CI: 1.30, 3.97) times more likely to have had a favorable attitude towards antenatal care uptake compared to those women who didn't have media exposure **(Table 6).**

Discussion

The present study assessed comprehensive knowledge of antenatal care, attitude towards its uptake, and associated factors among home-delivered women in rural Sehala Seyemit district, northern Ethiopia. More than half of women had adequate knowledge of antenatal care, and about three-fourths of them had a favorable attitude towards its uptake.

The findings from this study were lower than previous studies conducted in Saudi (24), Nigeria (4), Libya (28), Ghana (4), and other researches in Ethiopia, including Mizan (3) and Fiche towns (23). This inconsistency might be because of differences in the study period, socio-demographic-characteristics, study population, and study setting. This study includes the rural population in which the habit of following health-related information and the level of recognition is lower. As supported by another study, rural women were less likely to use maternal health services due to limited access to information (33). Besides, the disparity may be a result of the tool we used to measure the outcome variable; most of the previously conducted researches measured the outcome variable using a few yes/no questions.

Women's knowledge of antenatal care was, however, higher than findings from Malaysia (34) and Nigeria (35). We might expect the disparity is differences in socio-demographic characteristics and the time gap. An alternative justification might be efforts have been made to enhance women's knowledge of any healthcare-related services in Ethiopia.

The current study has shown that the attitude of women towards antenatal care uptake was 75.2%, which was lower than findings in Libya (28) and Saudi (24). This inconsistency might be due to socio-demographic differences like educational status and residence. Hence the studies from Libya and Saudi reported that over two-thirds and more than 90% of the participants were secondary and above by education. However, almost all the study participants had no formal education in the current study. Evidences showed that educated women were more likely to use maternal healthcare services (4,17). On the other hand, in the present study, attitude towards antenatal care was higher than previous studies conducted in Nigeria (35) and Mizan town, Ethiopia (3). The variation might be for the time gap and increased awareness of women about maternal health services over time.

This study determined that the odds of adequate knowledge of ANC in the age groups of 20-30 and above 30 years old were seven and two times higher compared to those women less than or equal to 20 years old, respectively. This finding contradicts a previously

conducted study in Ghana in which older women were less likely to have had adequate knowledge of antenatal care (4). This discrepancy might be a difference in the study population in which we gathered the data. Evidence has determined that older maternal age is associated with maternal healthcare service utilization (17). Subsequently, if older maternal age enhances the practice of attending healthcare services, women may get knowledge concerning maternal and children's health.

Media exposure is an important predicting factor for women to have sufficient knowledge of antenatal care. Women who had mass media exposure were nearly four times more likely to be knowledgeable compared to those women who hadn't been exposed to mass media. The possible justification could be the health of women and children is a prime concern both at the national and global levels in which maternal and children's health-related information may have been given through media. Evidences also support that exposure to mass media increases the likelihood of maternal health service utilization (36,37).

This study likewise found that those women who traveled less than one hour to reach health facilities were 4.58 more likely to have had good knowledge of antenatal care compared to those women who traveled over an hour. The possible explanation could be women far from health facilities are less likely to use healthcare services since they became tired because of the long travel. Besides, the far residence from health institutions may deter health-seeking information, less chance of getting health professional advice and education. Studies have shown that longer distance impedes maternal and children's healthcare service utilization (38,39). The current study indicated that women who experienced obstetric danger signs in their recent pregnancy were seven times more likely to be knowledgeable compared to their counterparts. Finding supports that having ANC visits increase women's knowledge of obstetric danger signs (40). Again, women with a history of obstetric danger signs may visit health facilities to receive treatment, and realizing the consequences can be dangerous, as a result, they can hear more about the danger signs of pregnancy and antenatal care.

Similarly, the odds of having adequate knowledge of antenatal care were 11.6 times among women who had a history of abortion compared to those women who haven't had an abortion. If a woman has had an abortion before, she will likely have adequate antenatal care for the worry of recurrence and better outcomes of the current pregnancy.

Our finding found that women who had adequate knowledge of ANC were 2.2 times more likely to have had a positive attitude towards antenatal care uptake. Knowledgeable women have a better understanding of maternal health services and the bad outcomes of not using them. It is because knowledge is a roadmap for any process and act in healthcare practice.

Household decision-making power is another significant factor associated with a favorable attitude towards antenatal care uptake. Women who had higher decision-making power were eight times more likely to have had a positive attitude towards antenatal care uptake compared to women who had less decision-making power. If women are involved in any decision-making process, they are highly likely to use maternity and children's health services, so the knowledge and attitudes of women will be ideal. Research has shown that women's decision-making power strengthens the uptake of maternal health services (41) and women's good practices for neonatal illness (42).

The present study established that the likelihoods of retaining a favorable attitude towards antenatal care uptake among women who were assisted by HEWs for their recent delivery were 2.3 times compared to women who were assisted by family members. Even if they gave birth at home, women who are assisted by HEWs might have good advice about reproductive health, antenatal care, and general health services. Studies implicate that HEWs play an outstanding role in maternal and child healthcare service utilization. They refer women to the health facilities, assist delivery at home, and request for ambulances during the need for referrals (43,44).

Lastly, this study has shown that a favorable attitude towards antenatal care is significantly associated with mass media exposure. Those women who had mass media exposure were two times more likely to have a favorable attitude towards antenatal care uptake compared to women who didn't have mass media exposure. Media exposed women can further inquire about information to maternal and children's health and gain sufficient data regarding antenatal care and any family health. Furthermore, they can weigh the pros and cons, use maternal health services, and have a positive perspective.

Conclusion

Despite having an optimum attitude towards antenatal care uptake, the majority of women had insufficient knowledge of antenatal care. Older age, media exposure, less travel time to reach the health facility, history of abortion, and women who experienced obstetric danger signs were predictors of knowledge of antenatal care. Likewise, adequate

knowledge of ANC, higher household decision-making power, mass media exposure, and delivery assisted HEWs were factors independently associated with a favorable attitude of women towards ANC uptake.

Thus, integrated work is imperative to strengthen household decision-making power, mass media utilization, and arranging transportation access to raise women's knowledge of antenatal care and attitude towards its uptake, accordingly, to increase maternal and children's healthcare utilization.

Limitation and Strength of the Study

Social desirability bias may have been introduced, as the response is self-reported. The use of a probability sampling technique, adequate sample size, and community-based study could be the strength of this study.

Acknowledgments

We would like to thank Debre Berhan University for providing the study ethical clearance. Our gratitude also goes to all data collectors and study participants. We are glad to Sehala Seyemit Woreda Health Office for writing the permission letter.

Authors' Contributions

Conceptualization: Azmeraw Ambachew Kebede

Data curation: Azmeraw Ambachew Kebede, Birhan Tsegaw Taye, Kindu Yinges

Formal analysis: Azmeraw Ambachew Kebede, Birhan Tsegaw Taye, Kindu Yinges Wondie

Investigation: Azmeraw Ambachew Kebede

Methodology: Azmeraw Ambachew Kebede, Birhan Tsegaw Taye, Kindu Yinges

Validation: Azmeraw Ambachew Kebede, Birhan Tsegaw Taye, Kindu Yinges

Visualization: Azmeraw Ambachew Kebede, Birhan Tsegaw Taye, Kindu Yinges Wondie

Writing-original draft: Azmeraw Ambachew Kebede, Birhan Tsegaw Taye, Kindu Yinges Wondie

Writing- review and editing: Azmeraw Ambachew Kebede, Birhan Tsegaw Taye, Kindu Yinges

Data sharing statement

The datasets collected and analyzed for this study are available from the corresponding author and can be attained on a reasonable request.

Funding

The authors received no specific funding for this research.

Disclosure

The authors declare that they have no conflict of interest in this work.

Abbreviations

AOR, adjusted odds ratio; ANC, antenatal care; CI, confidence interval; COR, crude odd ratio; HEWs, health extension workers; SPSS, statistical package for social science; TBA, traditional birth attendants; WHO, world health organization.

References

1. WHO recommendations on maternal health guidelines approved by the WHO guidelines review committee.Geneva: World Health Organization; 2017 (WHO/MCA/17.10). Licence: CC BY-NC-SA 3.0 IGO.

2. Tekelab T, Chojenta C, Smith R, Loxton D. Factors affecting utilization of antenatal care in Ethiopia : A systematic review and meta- analysis. PLoS One. 2019;14(4):1–24.

3. Andualem Henok, Hamelmal Worku, Hana Getachew HW. Knowledge , Attitude and Practice of Antenatal Care Service among Married Women of Reproductive Age Group in Mizan Health Center , Southwest Ethiopia. J Med Physiol Biophys. 2015;16.

4. Afaya A, Azongo TB, Dzomeku VM, Afaya A, Salia SM, Adatara P, et al. Women ' s knowledge and its associated factors regarding optimum utilisation of antenatal care in rural Ghana : A cross- sectional study. PLoS One. 2020;15(7):1–19.

5. Abdulrida H, Hassan R, Sabri M. Knowledge and health-seeking practices of mothers attending primary health-care centers in baghdad al-karkh sector about danger signs in newborns. Mustansiriya Med J. 2018;17(1).

6. Tadesse Tilahun and Makeda Sinaga. Knowledge of obstetric danger signs and birth preparedness practices among pregnant women in rural communities of Eastern Ethiopia. Int J Nurs Midwifery. 2016;8(1):1–11.

7. Bishanga DR, Drake M, Kim Y-M, Mwanamsangu AH, Makuwani AM, Zoungrana J, et al. Factors associated with institutional delivery: Findings from a cross-sectional study in Mara and Kagera regions in Tanzania. PLoS ONE. 2018;13(12).

8. Fekadu GA, Kassa GM, Berhe AK, Muche AA, Katiso NA. The effect of antenatal care on use of institutional delivery service and postnatal care in Ethiopia : a systematic review and meta-analysis. BMC Health Serv Res. 2018;18(577):1–11.

9. Trends in maternal mortality 2000 to 2017: estimates by WHO, UNICEF, UNFPA, World Bank Group and the United Nations Population Division. Geneva: World Health Organization; 2019: Licence: CC BY-NC-SA 3.0 IGO.; 2017.

10. Tuncßalp Ö, Pena-Rosas JP, Lawrie T, Bucagu M, Oladapo OT, Portela A, Metin G€ulmezoglu A. WHO recommendations on antenatal care for a positive pregnancy

experience—going beyond survival. BJOG 2017; DOI: 10.1111/1471-0528.14599.

11. Zhao Q, Kulane A, Gao Y, Xu B. Knowledge and attitude on maternal health care among rural-to-urban migrant women in Shanghai , China. BMC Women ' s Heal. 2010;8:1–8.

12. Kinney M V, Kerber KJ, Black RE, Cohen B, Nkrumah F, Coovadia H. Sub-Saharan Africa ' s Mothers , Newborns , and Children : Where and Why Do They Die ? PLOS Med. 2010;7(6):1–9.

13. Centeral statical agency of Ethiopia and ICF. Ethiopian demographic and health survey. Addis Ababa, Ethiopia, and Rockville, Maryland, USA: CSA and ICF.2016.

14. Ethiopia Mini Demographic and Health Survey Key Indicators. Rockville, Maryland, USA: EPHI and ICF. 2019.

15. Woyessa AH, Ahmed TH. Assessment of focused antenatal care utilization and associated factors in Western. BMC Res Notes. 2019;12(277):1–7.

16. Kolola T, Morka W, Abdissa B. Antenatal care booking within the first trimester of pregnancy and its associated factors among pregnant women residing in an urban area: a cross- sectional study in Debre Berhan town, Ethiopia. BMJ Open. 2020;10:1–6.

17. Ayalew TW, Nigatu AM. Focused antenatal care utilization and associated factors in Debre Tabor Town , northwest Ethiopia. BMC Res Notes. 2018;11(819):1–6.

18. Dulla D, Daka D, Wakgari N. Antenatal Care Utilization and Its Associated Factors among Pregnant Women in Boricha District , Southern. 2017;14:76–84.

19. Terefe AN, Gelaw AB. Determinants of Antenatal Care Visit Utilization of Child-Bearing Mothers in Kaffa , Sheka , and Bench Maji Zones of SNNPR , Southwestern Ethiopia. 2019;6:1–11.

20. Gebremeskel F, Dibaba Y, Admassu B. Timing of First Antenatal Care Attendance and Associated Factors among Pregnant Women in Arba Minch Town and Arba Minch District , Gamo Gofa Zone , South Ethiopia. J Environ Public Health. 2015;7.

21. Tegegne TK, Chojenta C, Getachew T, Smith R, Loxton D. Antenatal care use in Ethiopia : a spatial and multilevel analysis. 2019;1–16.

22. Twg TWG. Proportion and Factors Associated with late Antenatal Care Booking

among Pregnant Mothers in Gondar Town , North West Ethiopia. 2015;19(June):94–100.

23. Shukure R, Simegnew T. Knowledge Towards Antenatal Care and Service Utilization Among Women in Fiche Town , North Shewa , Ethiopia. Am Jpornal Pediatrcis. 2018;4(1):1–5.

24. Alshabanah RF, Almohayya TS, Alahmari EM. Assessment of Knowledge , Attitudes and Practices of Expectant Mothers in Relation to Antenatal Care in Abha. 2018;70(January):939–42.

25. Yaya S, Uthman OA, Amouzou A, Ekholuenetale M, Bishwajit G. Inequalities in maternal health care utilization in Benin : a population based cross-sectional study. BMC Pregnancy Childbirth. 2018;18(194):1–9.

26. Hogan MC, Foreman KJ, Naghavi M, Ahn SY, Wang M, Makela SM, et al. Maternal mortality for 181 countries , 1980 – 2008 : a systematic analysis of progress towards Millennium Development Goal 5. Lancet. 2008;375(9726):1609–23.

27. Ononokpono DN, Odimegwu CO. Determinants of Maternal Health Care Utilization in Nigeria : a multilevel approach. PanAfrican Med J. 2014;17(Supp 1):1–6.

28. Ibrahim HK, El MD, Mohammed HO. Knowledge , attitude , and practices of pregnant women towards antenatal care in primary healthcare centers in Benghazi , Libya. 2014;119–26.

29. Adem N, Berhe KK, Tesfay Y. Awareness and Associated Factors towards Neonatal Danger Signs among Mothers Attending Public Health Institutions of Mekelle City, Tigray, Ethiopia. J Child Adolesc Behav. 2017;5(6).

30. Jennings L, Na M, Cherewick M, Hindin M, Mullany B, Ahmed S. Women ' s empowerment and male involvement in antenatal care : analyses of Demographic and Health Surveys (DHS) in selected African countries. 2014;1–11.

31. Zaman S Bin, Gupta R Das, Muhammed G, Kibria A, Hossain N. Husband ' s involvement with mother ' s awareness and knowledge of newborn danger signs in facility ‑ based childbirth settings : a cross ‑ sectional study from rural Bangladesh. BMC Res Notes. 2018;4–9.

32. Nigatu D, Gebremariam A, Abera M, Setegn T, Deribe K. Factors associated with women ' s autonomy regarding maternal and child health care utilization in Bale

Zone : a community based cross-sectional study. BMC Womens Health. 2014;14(79).

33. Ayele DZ, Belayihun B, Teji K, Ayana DA. Factors Affecting Utilization of Maternal Health Care Services in Kombolcha District , Eastern Hararghe Zone , Oromia Regional State , Eastern Ethiopia. Int Sch Res Not. 2014;2014(2001).

34. Rosliza, Muhamad. Knowledge , attitude and practice on antenatal care among orang asli women in jempol , negeri sembilan. 2011;11(2):13–21.

35. Ogunba BO. Knowledge and Attitude of Women and Its Influence on Antenatal Care Attendance in Southwestern Nigeria. J Nutr Heal Sci. 2020;4(2).

36. Ousman SK, Mdala I, Thorsen VC, Sundby J. Social Determinants of Antenatal Care Service Use in Ethiopia : Changes Over a 15-Year Span. Front Public Heal. 2019;7(161):1–10.

37. Basha GW. Factors Affecting the Utilization of a Minimum of Four Antenatal Care Services in Ethiopia. Obstet Gynecol Int. 2019;6.

38. Terefe W. Distance from health facility and mothers ' perception of quality related to skilled delivery service utilization in northern Ethiopia. Int J Women's Heal. 2017;9:749–56.

39. Tanou M, Kamiya Y. Assessing the impact of geographical access to health facilities on maternal healthcare utilization : evidence from the Burkina Faso demographic and health survey 2010. BMC Public Health. 2019;1–8.

40. Amenu G, Mulaw Z, Seyoum T, Bayu H. Knowledge about Danger Signs of Obstetric Complications and Associated Factors among Postnatal Mothers of Mechekel District Health Centers , East Gojjam Zone , Northwest Ethiopia. Hindawi J Sci. 2016;

41. Ghose B, Feng D, Tang S, Yaya S, He Z, Udenigwe O, et al. Women ' s decision-making autonomy and utilisation of maternal healthcare services : results from the Bangladesh Demographic and Health Survey. BMJ Open. 2017;1–8.

42. Kebede AA, Cherkos EA, Taye EB. Mother ' s Knowledge of Neonatal Danger Signs and Health-Seeking Practices and Associated Factors in Debretabor , Northwest Ethiopia : A Community-Based Cross-Sectional Study. Res reports Neonatol. 2020;47–58.

43. Region T, Jackson R, Tesfay FH, Godefay H. Health Extension Workers ’ and Mothers ’ Attitudes to Maternal Health Service Utilization and Acceptance in Adwa Woreda ,. 2016;1–15.

44. Jackson R, Hailemariam A. The Role of Health Extension Workers in Linking Pregnant Women With Health Facilities for Delivery in Rural and Pastoralist Areas of Ethiopia. Ethiop J Heal Sci. 2016;26.

Table 1: Socio-Demographic Characteristics of Study Participants in Rural Sehala Seyemit District, Northern Ethiopia, 2020 (n= 653)

Characteristics	Category	Frequency	Percentage (%)
**Age of women in year**	≤20	70	10.7
	21-30	440	67.4
	≥31	143	21.9
**Current marital status**	Married	635	97.2
	Unmarried	18	2.8
**Family size**	2-3	84	12.9
	4-6	433	66.3
	≥7	136	20.8
**Women's educational status**	Unable to read and write	384	58.8
	Able to read and write	254	38.9
	Primary education	15	2.3
**Women's occupation**	Famer	533	81.6
	Merchant	89	13.6
	Others [a]	31	4.8
**Husband educational status (n= 635)**	Unable to read and write	235	37
	Able to read and write	324	51
	Primary education and above	76	12
**Husband occupation (n= 635)**	Famer	540	85
	Merchant	92	14.5
	Others [a]	3	0.5
**Exposure to mass media**	Yes	433	66.3
	No	220	33.7
**Type of transport at the time of emergency**	On foot/ traditional ambulance	426	65.3
	Ambulance	142	21.7
	Public transport	85	13

Note: [a] - student and daily labor, Traditional ambulance- it is made of wood and is used to transport mothers to health facilities in areas where there is no car

Table 2: Reproductive and Maternity Healthcare Service Characteristics of Study Participants in Rural Sehala Seyemit District, Northern Ethiopia, 2020 (n= 653)

Characteristics	Category	Frequency	Percentage (%)
**Parity**	1	74	11.3
	2-4	436	66.8
	≥5	143	21.9
**Had ANC**	Yes	198	30.2
	No	455	69.8
**Number of ANC follow-up (n= 198)**	<4	191	96.5
	≥4	7	3.5
**Birth assistant**	HEWs	379	58
	TBA	204	31.3
	Family members	70	10.7
**Had postnatal care**	Yes	82	12.6
	No	571	87.4
**Husband involvement**	Involved	363	56.6
	Not involved	290	44.4
**History of abortion**	Yes	63	9.6
	No	590	90.4
**History of neonatal death**	Yes	19	2.9
	No	634	97.1
**Experienced obstetric danger signs**	Yes	119	18.2
	No	534	81.8
**Time to the health facility**	< 1 hour	326	49.9
	≥ 1 hour	327	50.1
**Household decision-making power**	Higher	495	75.8
	Lower	158	24.2
**Pregnancy status**	Planned	573	87.7
	Unplanned	80	12.3

Table 3: Comprehensive Knowledge of Antenatal Care among Home-Delivered Women in Rural Sehala Seyemit District, Northern Ethiopia, 2020 (n= 653)

**Variables**	**Frequency**	**Percentag (%)**
Ever heard about ANC	Yes (556)<br>No (97)	85.1<br>15.9
Starting early ANC is important	Yes (367)<br>No (286)	56.2<br>43.8
Pregnant women may have problems without ANC	Yes (402)<br>No (251)	61.6<br>38.4
ANC has to be recommended regardless of complications	Yes (382)<br>No (271)	58.5<br>41.5
Maternal waiting homes are important in area's far from a health facility	Yes (357)<br>No (296)	54.7<br>45.3
Health facility delivery is safer and better than home delivery	Yes (534)<br>No (119)	81.8<br>18.2
Regular ANC medications can promote optimal growth of the unborn fetus	Yes (426)<br>No (227)	65.2<br>34.8
ANC can prevent complications	Yes (452)<br>No (201)	69.2<br>30.8
Alcohol drinking during pregnancy is bad for the fetus	Yes (329)<br>No (324)	50.4<br>49.6
Smoking during pregnancy is unsafe for the fetus	Yes (585)<br>No (68)	96.9<br>3.1
Do you know when to start ANC	3 to 4 months (362)<br>4 to 5 months (90)<br>Before 3 months (23)<br>Don't know (178)	55.4<br>13.8<br>3.5<br>27.3
Perception of first fetal movement	In the 3rd month (23)<br>Between the 4th and 5th months (271)<br>Don't know (359)	3.5<br>41.5<br>55
At which stage of pregnancy fetal deformities most likely occur?	Below 3rd month (124)<br>Between the 3rd and the 7th months (92)	19<br>14.1

	After the 7th month (12)<br>Don’t know (425)	1.9<br>65
The recommended number of ANC	Two (62)<br>Three (183)<br>Four (117)<br>Don’t know (291)	9.5<br>28<br>17.9<br>44.6
Prevention of malaria during pregnancy	Use of insecticide-treated nets (201)<br>Use of antibiotics (17)<br>Avoid stagnant water (28)<br>Close door and window (21)<br>Don't know (386)	30.8<br>2.6<br>4.3<br>3.2<br>59.1
Prevention of anemia during pregnancy	Avoid coffee, tea, and milk with a meal (29)<br>Use of routine iron (147)<br>Diet containing green and leafy vegetables (35)<br>Diet containing red meat and liver (24)<br>Don’t know (418)	4.5<br>22.5<br>5.3<br>3.7<br>64
Prevention of tetanus during pregnancy	Tetanus toxoid vaccine (194)<br>Don’t know (459)	29.7<br>70.3
Prevention of intestinal parasitic infection during pregnancy	Use of mebendazole (179)<br>Avoid barefoot (23)<br>Avoid eating raw meat (54)<br>Don’t know (397)	27.4<br>3.5<br>8.3<br>60.8
Knowledge of obstetric danger signs during pregnancy	Vaginal bleeding<br>Yes (402)<br>No (251)<br>Severe headache<br>Yes (220)<br>No (433)<br>Convulsion<br>Yes (176)<br>No (477)<br>Loss of consciousness<br>Yes (307)	 61.6<br>38.4<br><br>33.7<br>66.3<br><br>26.9<br>73.1<br><br>47

	No (346)	53
	Epigastric pain	
	Yes (240)	36.8
	No (413)	63.2
	Pre-labor rupture of membrane	
	Yes (385)	58.9
	No (268)	41.1
	Facial or upper extremity edema	
	Yes (250)	38.3
	No (403)	61.7
	Decreased fetal movement	
	Yes (348)	53.3
	No (305)	46.7
	Blurring of vision	
	Yes (180)	27.5
	No (473)	72.5
Knowledge of birth preparedness and complication readiness plan	Identify qualified birth attendants (12)	1.8
	Identify health facility for delivery (23)	3.5
	Arranged transport for emergency (86)	13.3
	Saving money (121)	18.5
	Prepare blood donor (4)	0.6
	Prepare materials for delivery (105)	16.1
	Identify danger signs and ready complication (19)	2.9
	I don't know (283)	43.3
What complications a woman will face without ANC?	Abortion (178)	27.3
	Intrauterine fetal death (156)	23.9
	Maternal complications (41)	6.3
	Don't know (278)	42.5
Overall comprehensive knowledge of ANC	Adequate knowledge (369)	56.5
	Inadequate knowledge (284)	43.5

Table 4: Attitude towards Antenatal Care Uptake by Component among Home-Delivered Women in Rural Sehala Seyemit District, Northern Ethiopia, 2020 (n= 653)

**Variables**	**Strongly agree (%)**	**Agree (%)**	**Neutral (%)**	**Disagree (%)**	**Strongly disagree (%)**
Want to have ANC follow up for next time	158 (24.2)	295 (45.1)	121 (18.5)	48 (7.4)	31(4.8)
Intention to deliver in a health facility for the next pregnancy	85 (13)	280 (42.9)	108 (16.5)	105 (16.1)	75 (11.5)
Health care professionals providing antenatal care is good	32 (4.9)	382 (58.6)	118 (18)	84 (12.8)	37 (5.7)
All pregnant women should have ANC follow up	221(33.8)	260 (39.8)	88 (13.5)	53 (8.1)	35 (5.4)
Timely ANC follow up will be safer for both mother and baby	145 (22.2)	305 (46.7)	82 (12.6)	95 (14.5)	26 (4)
Want to pay for ANC if it is with fee	76 (11.6)	186 (28.5)	91 (13.9)	180 (27.6)	120 (18.4)
Husbands should be present during ANC follow-up	148 (22.7)	370 (56.6)	64 (9.8)	41 (6.3)	30 (4.6)
**Advice regarding proper health during pregnancy can be gotten outside the hospital**	34 (5.2)	154 (23.6)	290 (44.4)	105 (16.1)	70 (10.7)
Follow up during pregnancy may decrease antenatal and postnatal complications	135 (20.7)	333 (51)	85 (13)	62 (9.5)	38 (5.8)
Overall attitude	Favorable (491)<br>Unfavorable (162)				75.2%<br>24.8

Table 5: Bi-Variable and Multivariable Logistic Regression Analysis of Factors Associated with Knowledge of Antenatal Care among Home-Delivered Women in Rural Sehala Seyemit District, Northern Ethiopia, 2020 (n=653)

**Variables**	**Category**	**Knowledge of women**		**COR (95%CI)**	**AOR (95%CI)**
		**Adequate**	**Inadequate**		

**Age in year**	≤ 20	31	39	1	1
	21-30	219	221	1.25 (0.75, 2.07)	2.26 (1.73, 6.15) *
	>31	119	24	6.24 (3.3, 11.9)	7.2 (3.43,15.1) *
**Women's occupation**	Farmer	286	247	1	1
	Merchant	74	15	4.26 (2.39, 7.61)	1.89 (0.78, 3.47)
	Others [a]	9	22	0.35 (0.16, 0.78)	1.15 (0.46, 2.68)
**Exposure to media**	Exposed	297	136	4.49 (3.17, 6.35)	3.69 (2.41, 5.65) **
	Not exposed	72	148	1	1
**Parity**	1	37	37	1	1
	2-4	220	216	1.02 (0.62, 1.67)	1.12 (0.16, 7.74)
	>4	112	31	3.61 (1.97, 6.62)	2.94 (0.26, 3.94)
**Family size**	2 -3	41	43	1	1
	4-6	219	214	1.07 (0.67, 1.71)	1.3 (0.7, 2.43)
	≥7	109	27	4.23 (2.32. 7.72)	0.69 (0.29, 1.63)
**History of abortion**	Yes	70	9	7.54 (5.6, 9.6)	11.6 (3.3, 14.6) *
	No	290	281	1	1
**Time to reach health facility**	<1 hour	237	194	3.87 (2.88, 5.37)	4.58 (3.05, 6.88) *
	≥ 1 hour	132	90	1	1
**Experienced danger signs**	Yes	99	20	4.84 (2.9, 8.1)	7.3 (3.9, 13.6) **
	No	270	264	1	1
**Decision-making power**	Higher	314	181	3.25 (3.23, 4.73)	1.17 (0.68, 1.98)
	Lower	55	103	1	1

Notes: [a] = student and daily laborer, * P≤0.05, **P ≤0.001

Abbreviations: AOR, adjusted odds ratio; COR, crude odds ratio; CI, confidence interval; 1, reference category

Table 6: Bi-Variable and Multivariable Logistic Regression Analysis of Factors Associated with Attitude towards Antenatal Care Uptake among Home-Delivered Women in Rural Sehala Seyemit District, Northern Ethiopia, 2020 (n=653)

Variables	Category	Attitude of women		COR (95%CI)	AOR (95%CI)
		Favorable	Unfavorable		
**Age in year**	≤ 20	62	8	1	1
	21-30	309	131	0.3 (0.14, 0.65)	0.95 (0.39, 2.3)
	>31	120	23	0.67 (0.28,1.59)	0.56 (0.2, 1.54)
**Husband involvement**	Involved	316	47	4.42 (3.0, 6.5)	1.7 (0.77, 3.74)
	Not involved	175	115	1	1
**Knowledge of ANC**	Adequate	329	40	6.2 (4.14, 9.27)	2.2 (1.26, 3.71) **
	Inadequate	162	122	1	1
**Decision-making power**	Higher	436	59	13.84 (9.04,21.2)	8.3 (4.8, 13.83) *
	Lower	55	103	1	1
**History of danger signs**	Yes	108	11	3.87 (2.0, 7.4)	2.36 (1.04, 5.37)
	No	383	151	1	1
**Delivery assistant**	HEWs	256	128	2.24 (1.14, 4.4)	2.3 (1.1, 5.1) **
	TBA	225	29	0.21 (0.11, 0.41)	0.64 (0.29, 1.41)
	Family	10	5	1	1
**Women's education**	Unable to read and write	256	128	1	1
	Able to read and write	225	28	3.88 (2.49, 6.03)	1.43 (0.73, 2.88)
	Primary education	10	5	1.0 (0.33, 2.99)	0.61 (0.15, 2.52)
**Media exposure**	Yes	386	47	8.99 (6.0, 13.4)	2.27 (1.30, 3.97) **
	No	105	115	1	1

Notes: * P≤0.05, ** P ≤0.001

Abbreviations: AOR, adjusted odds ratio; COR, crude odds ratio; CI, confidence interval; HEWs, health extension workers; 1, reference category

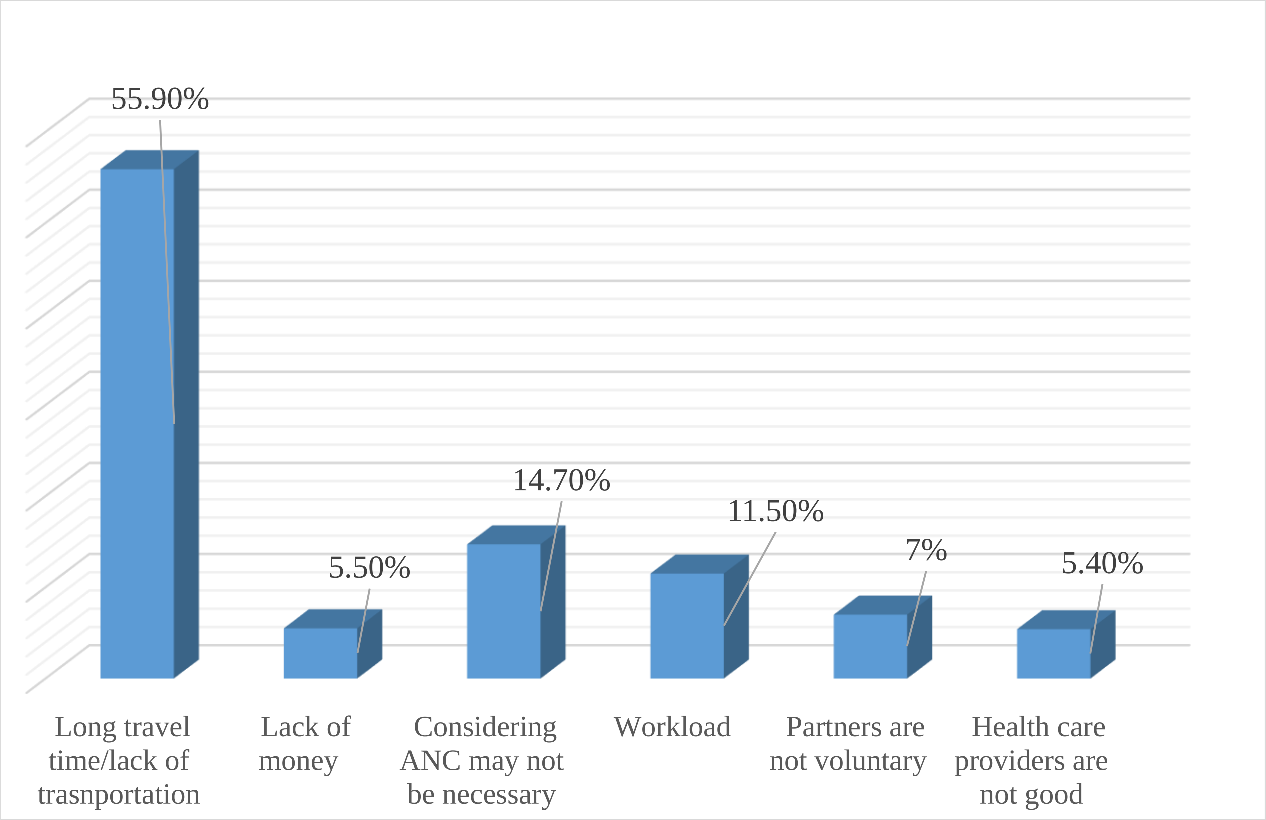

Figure 1: Barriers to Use Antenatal Care and Institutional Delivery among Home-Delivered Women in Rural Sehala Seyemit District, Northern Ethiopia, 2020.
