# Supplementary material for: Factors associated with comprehensive knowledge of antenatal care and attitude towards its uptake among women delivered at home in rural Sehala Seyemit district, northern Ethiopia: A community-based cross-sectional study
Source: PLoS One. 2022 Oct 12;17(10):e0276125. doi: 10.1371/journal.pone.0276125 (PMC9555639; doi:10.1371/journal.pone.0276125)
Supplement: Attachment — Submitted filename: Review_.Ethiopia_Knowledge_ANCdocx.docx [file pone.0276125.s004.docx]

**Review comments:**

I think, in general, the study is very interesting, providing important issues about maternal attitudes towards ANC, their knowledge, partner involvement. The authors highlighted crucial factors about seeking ANC care for women who delivered at home, the results were nicely reported, and discussion was in accordance with the aims. I have some comments, which are presented below:

1. It would be helpful for reader to understand the study population, if the authors described them in terms of general Ethiopia population – how do they differ from general population, if there is any specific characteristics which might provide different results, any factors affecting specifically study population?

It was mentioned in the manuscript p. 6. ‘Before the actual data collection, we did a pretest on 34 women at Ziquala Woreda which has similar sociocultural and living standards as the study population.’ – that’s good. Just to add probably one paragraph about general vs study population.

2. As mentioned in the methods section, all home deliveries are included in the study, and if I understood correctly, you chose 670 women among those who delivered home (and did not have complications)? If so, what is this proportion? How many were women who gave birth in recent two years, how many among those delivered at home and how many did not have complications?
3. It would be also nice if authors can include some information about financing ANC and delivery-related costs, how it works in the country? Every pregnant woman can have free 4 visits (as it says they still have 4 ANC visits rather than 8), is it free of charge or do they need to pay some part of it? Also, is facility-based delivery paid out-of-pocket or by government?
4. The aim of the study was: ‘The present study assessed comprehensive knowledge of antenatal care, attitude towards its uptake, and associated factors among home-delivered women in rural Sehala Seyemit district, northern Ethiopia.’ I think, the first two aims are fine, I have concerns about the third aim – associated factors among home-delivered women – for me, its more relevant to say when comparing women who delivered at home to those who delivered at the institution, but in this study only women who delivered at home were included. I would suggest modifying the last aim slightly and address it in a way like you have presented in the results, probably including ‘barriers to use institutional deliveries’ or something similar.
5. In discussion part it is mentioned that: ‘This inconsistency might be because of differences in the study period, socio-demographic-characteristics, study population, and study setting’ – that’s exactly my concern – how does this study population differ from general population in terms of socio-demographic characteristics, if you could add the information about it, probably in the methods section when describing study population.
6. I suggest explaining strengths and limitations in more details.
