# Supplementary material for: Factors associated with comprehensive knowledge of antenatal care and attitude towards its uptake among women delivered at home in rural Sehala Seyemit district, northern Ethiopia: A community-based cross-sectional study
Source: PLoS One. 2022 Oct 12;17(10):e0276125. doi: 10.1371/journal.pone.0276125 (PMC9555639; doi:10.1371/journal.pone.0276125)
Supplement: Attachment — Submitted filename: Response to Reviewers.docx [file pone.0276125.s005.docx]

December 7, 2021

Manuscript ID: PONE-D-21-01426

Title: Factors associated with comprehensive knowledge of antenatal care and attitude towards its uptake among home-delivered women in rural Sehala Seyemit district, northern Ethiopia: a community-based cross-sectional study

Journal: PLoS ONE

First of all, we would like to thank the reviewers and the editor for their constructive comments and suggestions for the significant improvements to the manuscript. We found all the comments and suggestions were beyond crucial. Thus, without hesitation, we have incorporated almost all the suggestions and comments in the revised manuscript. The changes made during revision have been shown with tracked changes and a point-by-point response is written below-please see below.

Response to Reviewers’ comments

Reviewer #1

| S.N | Comments and/or questions | Responses |
| --- | --- | --- |
| 1 | The Authors have done a good work on this important area of antenatal care. However, there are minor corrections to consider. | Thank you, dear reviewer for your time and important feedback for the improvements of our manuscript. |
| 2 | Table 1, Education status for the woman and husband should be in meaningful educational levels. Someone either know how to read and write or does not know. Primary education is a meaningless category there. | Thank you, dear reviewer. A person may able to read and write without attending formal education. However, primary education (1-8 grade in Ethiopian context) in this case implies that there are people who have attend formal education (attending education based the national curriculum). |
| 3 | The variable, "type of transport at the time of emergency" typically apply when health facilities or locations are studied or when the study dwells on transport type available to women who experienced an emergency. As it applies here all participants from the same location should ideally give the same answer. | Thank you, dear reviewer. Yes, you are right, all the study populations were living in rural area and will have almost similar answer. However, there some kebeles, in which traveling on foot to reach health facility is impossible. On the other hand, there are some rural kebeles close to health facilities and women may not need an ambulance. So, it depends on the distance of the kebele to the health facility. |
| 4 | Study limitations should include an inherent bias in memorizing due to the retrospective nature of the study | Thank you, dear reviewer. We have incorporated it in the revised manuscript. Please see the limitation in the revised manuscript. |
| 5 | Table 3 looks like containing raw data. It can be summarized with analytical data. This current table can be uploaded as additional data file. | Thank you, dear reviewer. We put the summarized analytical data in Table 3 and the previous table has been uploaded as a supportive file with its detail measurement as per you kind recommendation. Please kindly see the revised manuscript. |

Reviewer #2

| S.N | Comments | Responses |
| --- | --- | --- |
| 1 | In line with the study objectives and the scope of the study, I would recommended the author revise the title to “Factors Associated with Comprehensive Knowledge of Antenatal Care and Attitude Towards ANC Uptake among Home-Delivered Women In Rural Sehala Seyemit District, Northern Ethiopia: A Community-Based Cross Sectional Study | Thank you, dear reviewer, for your time and commitment you made for the significant improvement of this manuscript. We did amendment to the title based on your kind recommendation. |
| 2 | Abstract…. Introduction: you need to tighten your introduction. Transition from the first sentence to the second is weak. You talked about Ethiopia having the highest Maternal Mortality then straight to the gap of your study. | Thank you, dear reviewer. We tried to make it strong with good transition. Please kindly take a look at the revised manuscript. |
| 3 | Abstract…..  Methodology: Significant variables are not determined by adjusted odds ratio. Revise this sentence “Statistically significant association of variables was determined based on adjusted odds ratio with a 95% confidence interval and p-value of ≤0.05”. In addition, the word statistically significant is tautology. You can use “significantly associated”. Change bivariable to bivariate and multivariable to Multivariate. Do the same in the main work. | Thank you, dear reviewer, for your careful and deep reading. All the comments have been taken positively and incorporated in the revised manuscript. However, as we tried to review evidence, there is a bit difference between bivariable/bivariate and multivariable/multivariate. The terms multivariate and multivariable are often used interchangeably in the public health literature. However, multivariate implies a statistical analysis with multiple outcomes. In contrast, multivariable analysis is a statistical tool for determining the relative contributions of various factors to a single event or outcome. Please kindly visit the following websites/articles:  1: Distinction between Two Statistical Terms: Multivariable and Multivariate Logistic Regression: doi:10.1093/ntr/ntaa055.  2: [Multivariable analysis: a practical guide for clinicians and public health researchers](https://books.google.it/books?hl=en&lr=&id=-X4G4dHsARQC&oi=fnd&pg=PR1&dq=Multivariable+Analysis+A+Practical+Guide+for+Clinicians+and+Public+Health+Researchers&ots=XDptnmBC6e&sig=rA68T2_H_IcQA4VxkA-RAZdduhM).  3: Multivariable Analysis in Cerebrovascular Research: Practical Notes for the Clinician: DOI: 10.1159/000345491 etc. Please kindly take the other changes in the revised manuscript. |
| 4 | Abstract  Results: Please take following out from the first sentence (95% CI; 52.6, 60.6), (95% CI; 71.8, 78.5). Is a univariate reporting and this does not need them. They are more relevant for multivariate reporting. | Thank you, dear reviewer. It has been removed in the revised manuscript. |
| 5 | **Introduction:**  The introduction was well written, however, they need to revise the introduction to include the context of home delivery. Home delivery which is a major part of the work was mentioned once in the introduction (paragraph 7). The introduction should capture the main ideas of the work; ANC knowledge, attitude towards ANC uptake and home-based delivery | Thank you, dear reviewer. We tried to revise the introduction section based on your kind recommendation. We did significant changes, including paragraph rearrangements in the introduction section. Please see the revised manuscript. |
| 6 | Introduction…..  I believe the study population (home based delivery respondent) has an impact on the findings of the study. Please justify why you choose home-based population. Why is it important to study knowledge on antenatal care and attitude towards ANC uptake among women who delivered at home? | Thank you, we have tried to justify and incorporated in the revised manuscript |
| 7 | Introduction….  Paragraph 3, first sentence, kindly get a more recent statistics. 2017 global maternal mortality rate of 295, 000 is a bot old considering we are in 2021. The figure is almost 4 years and things have changed. Using 2019 or 2020 maternal mortality figure would be appropriate | Thank you. We tried to find a recent figure but the global figure of maternal mortality is the 2017. Of course, the data was went online in the year 2019, but the data describes the MMR from 2000 to 2017. (<https://www.un.org/en/development/desa/population/publications/pdf/mortality/WMR2019/WMR2019_Highlights.pdf>, Trends in maternal mortality 2000 to 2017: estimates by WHO, UNICEF, UNFPA, World Bank Group and the United Nations Population Division. Geneva: World Health Organization; 2019: Licence: CC BY-NC-SA 3.0 IGO. So, we are not able to find more latest data other than this. |
| 8 | Introduction….  Paragraph 7, first sentence, please delete “elsewhere” and be specific on the studies. Include the region or countries that the studies were conducted. In addition, less emphasized was put on home delivery in the problem statement. This is where the paper should elaborate on home delivery. | Thank you. All the comments were incorporated in the revised manuscript. |
| 9 | Introduction…  Please revise the last paragraph and highlight the new thing(s) that the study intends to highlight. | Thank you. We tried to highlight the gaps what this study tried to fill. |
| 10 | **Methodology**  Study designs, setting and period References should be provided to support some of the claims. For instance, there should be reference for the “total population” and the sentence “accessing health services in the district is difficult because of the lack of transportation to each “kebeles”. | Thank you, dear reviewer. From the beginning we tried to find a published data. However, we didn’t find any published data, rather we have obtained unpublished documented data from the Woreda and we have mentioned it previously in this section. |
| 11 | Methodology….  Sample size determination and sampling procedure Women’s attitude towards ANC of 70.6% is from where? Be specific if it is a district figure or a national figure | Thank you, dear reviewer. It is a district figure in Ethiopia and included in the revised manuscript. Please kindly see the revised manuscript. |
| 12 | Methodology…  Please explain the steps involved in applying multistage sampling very well. What you have written is not clear to me. | Thank you, dear reviewer. In the 1^st^ stage, 8 kebeles were selected and the total number of women in the selected kebeles were obtained. In the 2^nd^ stage, 670 women were selected by using simple random sampling technique from the designed frame. From here, simple random sampling technique was used to select both the kebeles and the study participants. So, since it is two stage, it is possible to say multistage. Please kindly see the revised manuscript. |
| 13 | Methodology (Measurement of variables)…  Please include questions on the comprehensive knowledge of ANC. Please provide details on how the knowledge of antenatal care was categorized. Because some of the responses were binary (yes and no) whilst others were multi-response. How did you treat questions with three or four responses as well as the binary response to arrive at the adequate and inadequate responses? Explain the process of arriving at the categorization well. Provide justification for using the mean as the cutoff point for favorable/unfavorable and adequate/inadequate respectively. Is it because the data were skewed or it was symmetric or something else. | Thank you, dear reviewer. You raised very important and basic suggestions and questions. We have included the questions in the revised manuscript as supporting information and put the detail how the knowledge was measured and we did some amendments in the table presentation. Since it is detail, we have included it as a supporting file and uploaded separately (S1 file: Detail of knowledge measurement). In addition, we used the mean based on the previous findings as we cited in the mother document. In addition, the data were not skewed. Please kindly see the revised manuscript. |
| 14 | Methodology….  Is the Likert scale questions on women’s attitude an existing scale? If so, Kindly indicate the source and studies that have used the Likert scale. If not, provide the justification for using the Likert scale. | Thank you, dear reviewer. We used a Likert scale based questions based on the previous studies and we have cited there. In addition, empirical evidence recommend that Likert Scale is an ordinal psychometric measurement of attitudes, beliefs and opinions despite its social desirability bias (https://www.simplypsychology.org/likert-scale.html). The “Yes” or “No” response may not consider the different degree of agreements or disagreements, even may not consider those who are neutral. |
| 15 | Methodology …  Why is it that only two independent variables thus husband involvement and Household decision making power were explained. Kindly explain how the rest of the independent variables were measured. | Thank you, dear reviewer, you raised a very important question. We perceived that the other variable were easy to understand and were not measured using a composite variable. For now, we have taken your kind recommendation positively and after careful reading, we put a definition for variables like media exposure, experienced dangers signs, history of neonatal death, and history of abortion in the revised manuscript. Please kindly see the revised manuscript. |
| 16 | Methodology (Data collection and instruments)...  The first sentence should read “The data collection tool was developed by reviewing literature (3, 4, and 23). The data were collected using a semi-structured questionnaire through face-to-face interviews. | Thank you, dear reviewer, for your positive suggestion. We take it positively and corrected in the revised manuscript. |
| 17 | Methodology…  Please include a statement in the data collection and instruments on how participants consented to be part of the study. Was participants confidentiality and anonymity assured? If so, kindly be specific. | Thank you, dear reviewer. We have included it in the ethical consideration part- please kindly see the revised manuscript. |
| 18 | Methodology…  Data Processing and analysis The first paragraph, line 5 reads “Binary logistic regression analysis was fitted to identify statistically significant independent variables, and variables having a p-value of less than 0.2 were included in the multivariable logistic regression for controlling confounders”. Please explain why you used confidence level of 80%, thus p value less than 0.2 at the bivariate level. Binary logistic regression analysis should be changed to bivariate analysis. The type of regression run at the multivariate level should be specified. Kindly provide reasons why you used p value less than 0.2 at the bivariate level and p value less than 0.05 at the multivariate level. | Thank you dear reviewer for your concern. Please accept our apologies, it was typing error. The basis was to take all variables having a p-value of <0.25 to set for the multivariable regression. However, it was written < 0.2 and it was typing error. We did based on the assumption to include all variables having a p-value of <0.25 as evidenced by existing literature (<http://dx.doi.org/10.21037/atm.2016.02.15>, doi: 10.1186/1751-0473-3-17, doi: 10.1186/1751-0473-3-17). For this study, we used a p-value of 0.25 assumption to proceed to the multivariable logistic regression, however, all variables which fulfill the chi-square assumption had a p-value of ≤ 0.2. The principle we used has been corrected in the revised manuscript, in which even if all the variables had a p-value of ≤ 0.2, the standing assumption was to include variables having a p-value <0.25. In addition, we used 0.05 to declare significant association the level of confidence to accept or reject the null hypothesis was 95% and it is the widely used principle. Moreover, the Backward LR analysis was undertaken and it is included in the revised manuscript. |
| 19 | Ethical Approval and Consent to Participate The last sentence “Both oral and written informed consent was collected from each of the study members was obtained after a clear explanation of the aim of the study” should be revised. Add if all the respondent signed informed consent form or some thumbprint. | Thank you, dear reviewer. We have included your thoughtful suggestion in the revised manuscript. |
| 20 | **Results**  This sentence should be revised “Most (97.2%) of the participants were in marital union and 384 (58.8%) of them were unable to read and write by education”. Replace marital union with currently married and take away education at the end of the sentence. The sentence should now read “most (97.2%) of the participants were currently married and more than half (58.8%) of the participants were unable to read and write. | Thank you, dear reviewer. We have accepted the comments and corrected in the revised manuscript. |
| 21 | Show the p value for all the bivariate and multivariate variables. | Thank you, dear reviewer. We did that in the revised manuscript. |
| 22 | Please consider revising the tables by following this format. What you have presented is a bit confusing. The univariate and bivariate analysis should be together in one table and show the p value for each variable. This can be seen by having the variables, percentage (frequency), and then knowledge of women on ANC (adequate and inadequate). The multivariate table should be in one Table. | Thank you, dear reviewer. The bivariable and multivariable analysis were together. If we are not mistaken, for table 5 (regression table for knowledge) and table 6 (regression for attitude). So, in each table there is a column containing COR (showing the bivariable result) and AOR (showing the multivariable result). As we would understand so far, the bivariable and multivariable result should be in one table with its cross tabulation-what we have did. Moreover, we have included the p-values for both knowledge and attitude in the multivariable analysis. Dear reviewer, if there is any deviation from the journal requirement in this regard, the journal office will let we know. |
| 23 | From Table 1, on family size, I am wondering if all the women had at least 2 or more family size, Was there not any one with one family size? | Thank you, dear reviewer. We have got a minimum of 2 family size. There was no any woman with one family size. Only 18 women were unmarried. Fortunately, all of these women had children. |
| 24 | Change women’s educational level to women’s literacy because read and write are literacy indicators not education. The same with husband educational status | Thank you, dear reviewer. We have changed it in the revised manuscript. |
| 25 | On type of transport at the time of emergency, what is the difference between on foot, traditional ambulance, and ambulance? | Thank you, dear reviewer. All the study populations were living in rural areas. There are some kebeles, in which traveling on foot to reach health facility is impossible. On the other hand, there are some rural kebeles close to health facilities and women may not need an ambulance. Besides, there are some kebeles which have access to ambulance (most of the kebeles have no road for ambulance). So, it depends on the distance of the kebele to the health facility. Moreover, as we tried put a note below Table1, traditional ambulance- it is made of wood and is used to transport mothers to health facilities in areas where there is no transportation access (either lack of road access or car). Some others may went on foot if it is not too far. |
| 26 | The authors should run multicollinearity analysis | Thank you, multicollirity test was run from the beginning. Thus, the multicollinearity assumption was checked using the variance inflation factor (VIF), where VIF <10 was acceptable. Please-the revised manuscript. |
| 27 | Discussion  Second paragraph first sentence, please be clear on the specific findings that were lower than the previous studies Edit second paragraph. | Thank you, dear reviewer. It was to mean knowledge and corrected in the revised manuscript |
| 28 | Conclusion of the study From what I read in the manuscript, I think your conclusion is not sound. Again, what is the policy direction of your work, kindly suggest at least two policies from the findings of the study Recommend a future studies from your findings | Thank you, dear reviewer. We have included policy implications and recommendations for future researchers. |
| 29 | Limitation and strength of the study Aside social desirability, what are the other limitations of the study | Thank you, dear reviewer. We have included some limitations in the revised manuscript. Please kindly see the revised manuscript. |
| 30 | What is novel about your studies? What new thing are you adding to knowledge? Kindly make these clear. | Thank you, dear reviewer. Even if the topic is not truly novel, studies reaching out to women who gave birth at home is limited. In addition, most of the previously conducted studies regarding the proposed topic failed to apply logistic regression, they were simply descriptive. Thank you very much once gain for your time and commitment for the significant improvements of our manuscript. All of your comments and suggestions were beyond crucial for the manuscript improvement and almost all your comments were taken positively and incorporated in the revised manuscript. |

Reviewer #3

| S.N | Comments | Responses |
| --- | --- | --- |
| 1 | I think, in general, the study is very interesting, providing important issues about maternal attitudes towards ANC, their knowledge, partner involvement. The authors highlighted crucial factors about seeking ANC care for women who delivered at home, the results were nicely reported, and discussion was in accordance with the aims. I have some comments, which are presented below: | Thank you, dear reviewer. You did a great contribution for the improvements of this manuscript. |
| 2 | It would be helpful for reader to understand the study population, if the authors described them in terms of general Ethiopia population–how do they differ from general population, if there is any specific characteristics which might provide different results, any factors affecting specifically study population? It was mentioned in the manuscript p. 6. ‘Before the actual data collection, we did a pretest on 34 women at Ziquala Woreda which has similar sociocultural and living standards as the study population.’–that’s good. Just to add probably one paragraph about general vs study population. | Thank you, dear reviewer. There are no specific characteristics which makes this population unique from the other Ethiopian population. Of course, there may have socio-cultural and demographic differences. As we tried to point out from the study setting part, the geographic area is very difficult for transportation and healthcare access, even a woman may travel for more than 6-8 hours to reach a health facility. This situation may be common in other areas of Ethiopia. The sentence in p.6 implies that the two districts are more or less similar in all aspects including the language (ways of communication and accent), culture, geographical area, access to healthcare etc. |
| 3 | As mentioned in the methods section, all home deliveries are included in the study, and if I understood correctly, you chose 670 women among those who delivered home (and did not have complications)? If so, what is this proportion? How many were women who gave birth in recent two years, how many among those delivered at home and how many did not have complications? | Thank you, dear reviewer. Yes or course, you are right; 670 women were selected to be part of this study. As we have obtained data from the health extension workers and local administrators, there were a total of 923 women who gave birth at home. This magnitude might be higher before two years. Because nowadays, women are start using maternity waiting homes, thereby the institutional delivery rate is getting increased. However, home delivery is still higher since most of the populations are farmers. In addition, we were not clearly identifying women with complication. Because whether the women have experienced complication or not, we have included them for the current study. Because the primary objective of this study was to assess the knowledge and attitude of women regarding ANC and to identify the constraining factors to use maternal health services. A single sentence escribing the number of home-delivered women in the last two years has been added in the last section of study setting. Please see the revised manuscript. |
| 4 | It would be also nice if authors can include some information about financing ANC and delivery-related costs, how it works in the country? Every pregnant woman can have free 4 visits (as it says they still have 4 ANC visits rather than 8), is it free of charge or do they need to pay some part of it? Also, is facility-based delivery paid out-of-pocket or by government? | Thank you, dear reviewer. All maternal and neonatal health services (ANC, delivery, postnatal care, immunization etc. Are given for free in Ethiopia and no any payment for facility based delivery. The 4 ANC visit is still implemented; it is because Ethiopia still not adopt the WHO 2016 8 ANC recommendation due to some problems. Recently, however, some tertiary hospitals have been trying to adopt the recommendation, still it is not recommended in all over the country yet. A single sentence describing about financing maternal health services is added in the study setting section. Please see the revised manuscript. |
| 5 | The aim of the study was: ‘The present study assessed comprehensive knowledge of antenatal care, attitude towards its uptake, and associated factors among home-delivered women in rural Sehala Seyemit district, northern Ethiopia.’ I think, the first two aims are fine, I have concerns about the third aim – associated factors among home-delivered women – for me, and it’s more relevant to say when comparing women who delivered at home to those who delivered at the institution, but in this study only women who delivered at home were included. I would suggest modifying the last aim slightly and address it in a way like you have presented in the results, probably including ‘barriers to use institutional deliveries’ or something similar. | Thank you, dear reviewer, you raised very important questions. Yes, all the study population in this study were all women who gave childbirth at home. In this context, we wouldn’t expect all home-delivered women will have similar factors/reason for not use maternal health services. Because their knowledge level could be affected by many factors like their household decision making power, mass media exposure, consulting health extension works to assist their delivery at home, distance to reach health facility and many other. Besides, knowledge and attitude are factors for not to use maternal health services. The last one is additional objective, since the study population was home delivered women, we perceived that it is important to ask what the barriers to use maternal health services are. Whether they gave birth at home or at institutions (if we did the study from both population) the only variable we could add would be place of delivery. So, disregard to the place of delivery, women’s knowledge and attitude could be affected by many factors. We are grateful for your kind suggestion. |
| 6 | In discussion part it is mentioned that: ‘This inconsistency might be because of differences in the study period, socio-demographic-characteristics, study population, and study setting’ – that’s exactly my concern – how does this study population differ from general population in terms of socio-demographic characteristics, if you could add the information about it, probably in the methods section when describing study population. | Thank you, dear reviewer. Since socio-demography includes residence and education, we tried to explain the difference based on these variable. The study population in this study was rural and who gave birth childbirth at home. In this case, as cited by many scholars, being a rural dweller is main factors for not using institutional delivery and inadequate knowledge of maternal health services. On the other hand, the study population from the aforementioned studies were women coming for ANC. In this case, these women are already engaged in the services, so we would expected to have a higher level of knowledge. A further explanation is mentioned in the revise manuscript. Please see the revised manuscript and the track changes made. |
| 7 | I suggest explaining strengths and limitations in more details. | Thank you, dear reviewer. We tried describe the limitations and strengths in detail in the revised manuscript. Please kindly see the revised manuscript. |
