# Supplementary material for: Factors associated with comprehensive knowledge of antenatal care and attitude towards its uptake among women delivered at home in rural Sehala Seyemit district, northern Ethiopia: A community-based cross-sectional study
Source: PLoS One. 2022 Oct 12;17(10):e0276125. doi: 10.1371/journal.pone.0276125 (PMC9555639; doi:10.1371/journal.pone.0276125)
Supplement: Attachment — Submitted filename: My reviewer comments_1.docx [file pone.0276125.s006.docx]

**Abstract**

***Introduction:***

Comment: You need to be specific on the Sustainable development goal.

Kindy revise the statement “However, evidence regarding the hypothesized topic is scarce, particularly which addresses women who gave birth at home”. I am confused about the “hypothesized topic: which is hypothesis topic are you talking about here. Kindly go straight to the point and write for easy understanding.

Kindly reduce the background. It is too long

***Methodology***:

Comment: Kindly take pretested questionnaire from the sentence. The sentence should read “The data were collected using a semi-structured interview guide”

**Introduction**

**In paragraph 3, you argued that pregnant women should have at least eight ANC contacts or visit to antenatal clinics (**In 2016, the World Health Organization (WHO) recommends that all mothers should have at least eight ANC contacts to ensure better maternal and neonatal health) **but in paragraph 6 you argued about four visits (**In connection to this, there is a need to assess the knowledge and attitude of the conservative rural community towards ANC utilization to be in line with the WHO target, which says 90% of pregnant women should have at least four and above ANC contacts and give birth at health facilities by 2025)…. Kindly be consistent which is which???

**Last paragraph “you need to tighten your gap well. The paragraph does not read well and I do not really get the message, what is the contribution of the paper to literature and policy direction?**

Kindly specify the goal number for all the sustainable goals in the manuscript

**Methodology**

**Study population**

Kindly rewrite the sentence well. The sentence should read *“all women who gave birth in the last two years in the selected “Kebeles” during the data collection period were selected to be part of the study”*

***Measurement of variables***

Please provide details on how the knowledge of antenatal care was categorized. Because some of the responses were binary (yes and no) whilst others were multi-response. How did you treat questions with three or four responses as well as the binary response to arrive at the adequate and inadequate responses? Explain the process of arriving at the categorization well.

Is the Likert scale questions on women’s attitude an existing scale? If so, Kindly indicate the source and studies that have used the likert scale. If not, provide the justification for using the likert scale.

***Data Processing and analysis***

Kindly provide reasons why you used p value less than 0.2 at the bivariate level and p value less than 0.05 at the multivariate level. It is still not clear

**Results**

Please avoid using women and respondents interchangeably. Kindly use one

**Third sentence..change two-thirds of them to two-thirds of the respondents**

**Delete the full stop after “**Most (97.2%) of the study participants were currently married”

Kindly delete “(95 % CI; 52.6, 60.6)” from the section women’s comprehensive knowledge of ANC. Do the same for attitude towards ANC uptake.

**Conclusion of the study**

**I don’t understand this statement “**The results of this study are looking for relevant stakeholders and health policymakers who pay particular attention to women in rural areas, including advocation regarding maternal health services through mass media” The results are looking for who?? Please delete or rewrite

Your conclusion is still not sound.

There are grammatical errors in the work. Kindly edit the work.

Kindly rewrite this “Moreover, researchers should address the rural women, particularly those who gave childbirth at home and the reason for not using maternal health services should better be addressed through a qualitative study” How can researchers address
