# Supplementary material for: Factors associated with comprehensive knowledge of antenatal care and attitude towards its uptake among women delivered at home in rural Sehala Seyemit district, northern Ethiopia: A community-based cross-sectional study
Source: PLoS One. 2022 Oct 12;17(10):e0276125. doi: 10.1371/journal.pone.0276125 (PMC9555639; doi:10.1371/journal.pone.0276125)
Supplement: Attachment — Submitted filename: Response to Reviewers (R2).docx [file pone.0276125.s007.docx]

May 2, 2022

Manuscript ID: PONE-D-21-01426R1

Title: Factors associated with comprehensive knowledge of antenatal care and attitude towards its uptake among home-delivered women in rural Sehala Seyemit district, northern Ethiopia: a community-based cross-sectional study

Journal: PLOS ONE

First of all, we would like to thank the reviewers and the editor for their constructive comments and significant improvements to the manuscript. We found all the comments and suggestions were beyond crucial. We have incorporated all the suggestions and comments in the revised manuscript. The changes made during the revision have been shown with tracked changes and a point-by-point response-please is attached below.

Response to Reviewers’ comments

Reviewer #2

| S.N | Comments | Responses |
| --- | --- | --- |
| 1 | **Abstract**  ***Introduction:***  Comment: You need to be specific on the Sustainable development goal.  Kindy revise the statement “However, evidence regarding the hypothesized topic is scarce, particularly which addresses women who gave birth at home”. I am confused about the “hypothesized topic: which is hypothesis topic are you talking about here. Kindly go straight to the point and write for easy understanding. Kindly reduce the background. It is too long. | Thank you, dear reviewer, for your time and commitment you made for the significant improvement of the manuscript. We accepted all your kind suggestions and clearly put with track changes in the revised manuscript. |
| 2 | ***Methodology (abstract)***  Comment: Kindly take pretested questionnaire from the sentence. The sentence should read “The data were collected using a semi-structured interview guide”. | Thank you, dear reviewer. We made the correction based on your kind recommendation. Please see the changes in the revised manuscript. |
| 3 | **Introduction**  **In paragraph 3, you argued that pregnant women should have at least eight ANC contacts or visit to antenatal clinics (**In 2016, the World Health Organization (WHO) recommends that all mothers should have at least eight ANC contacts to ensure better maternal and neonatal health) **but in paragraph 6 you argued about four visits (**In connection to this, there is a need to assess the knowledge and attitude of the conservative rural community towards ANC utilization to be in line with the WHO target, which says 90% of pregnant women should have at least four and above ANC contacts and give birth at health facilities by 2025)…. Kindly be consistent which is which??? | Thank you, dear reviewer, for your thoughtful comment and careful reading. From the rough view, the ideas from the two paragraphs seem contradictory. In 2016, the WHO declared all pregnant women to have 8 ANC contacts. However, this recommendation is still not adopted by many developing countries. In connection to this, the WHO targets 90% of pregnant women should have at least four and above ANC contacts by 2025 towards increasing to 8 contacts by 2030. Therefore, the principle is, if possible all pregnant women should have 8 ANC contacts, if not at least 90% of pregnant women should have the previously recommended 4 and above ANC contact as a footpath to the 8 ANC contact. Please kindly visit the following references for clarity:  1. WHO recommendations on antenatal care for a positive pregnancy experience  2.https://www.who.int/news/item/05-10-2021-new-global-targets-to-prevent-maternal-deaths |
| 4 | Last paragraph “you need to tighten your gap well. The paragraph does not read well and I do not really get the message, what is the contribution of the paper to literature and policy direction?  Kindly specify the goal number for all the sustainable goals in the manuscript | Thank you, dear reviewer. We tried to do some revisions based on your kind recommendation. Please kindly see the revised manuscript which is shown by track changes. Besides, we put the specific SDG throughout the document. |
| 5 | **Methodology**  **Study population**  Kindly rewrite the sentence well. The sentence should read *“all women who gave birth in the last two years in the selected “Kebeles” during the data collection period were selected to be part of the study”* | Thank you, dear reviewer. Your kind recommendation has been taken without hesitation and clearly shown with track changes in the revised manuscript. |
| 6 | ***Measurement of variables***  Please provide details on how the knowledge of antenatal care was categorized. Because some of the responses were binary (yes and no) whilst others were multi-response. How did you treat questions with three or four responses as well as the binary response to arrive at the adequate and inadequate responses? Explain the process of arriving at the categorization well | Thank you, dear reviewer. You raised very important question. We have included the questions in the revised manuscript and put the detail of how the knowledge was measured and how the binary classification was made for the multiple responses. We put variables having multiple responses as correct and incorrect answers in the table. Since the detail is complex, we have included it as a supporting file and uploaded separately (S1 file: Detail of knowledge measurement). Please kindly visit the separately attached file as S1 file: Detail of knowledge measurement to see how the multiple responses were categorized in to a binary response. |
| 7 | Is the Likert scale questions on women’s attitude an existing scale? If so, Kindly indicate the source and studies that have used the Likert scale. If not, provide the justification for using the Likert scale | Thank you, dear reviewer. Yes, the Likert scale is an existing scale. We used a Likert scale based questions based on the previous studies and we have cited there (in the manuscript). In addition, empirical evidence recommend that Likert Scale is an ordinal psychometric measurement of attitudes, beliefs and opinions despite its social desirability bias (https://www.simplypsychology.org/likert-scale.html). The “Yes” or “No” response may not consider the different degree of agreement or disagreed, even may not consider those who are neutral. Therefore, we used an existing Likert scale and we have cited the references in the manuscript. |
| 18 | ***Data Processing and analysis***  Kindly provide reasons why you used p value less than 0.2 at the bivariate level and p value less than 0.05 at the multivariate level. It is still not clear | Thank you dear reviewer for your concern. Please accept our apologies, it was typing error. The basis was to take all variables having a p-value of <0.25 to set for the multivariable regression analysis. However, it was written < 0.2 and it was typing error. We did based on the assumption to include all variables having a p-value of <0.25 as evidenced by existing literature (<http://dx.doi.org/10.21037/atm.2016.02.15>, doi: 10.1186/1751-0473-3-17, doi: 10.1186/1751-0473-3-17). For this study, we used a p-value of 0.25 assumption to proceed to the multivariable logistic regression. The principle we used has been corrected in the revised manuscript, in which even if all the variables had a p-value of ≤ 0.2, the standing assumption was to include variables having a p-value <0.25. In addition, we used 0.05 to declare significant association at the multivariable analysis and the level of confidence to accept or reject the null hypothesis 95% and it is the widely used principle based on the abovementioned references. |
| 20 | **Results**  Please avoid using women and respondents interchangeably. Kindly use one | Thank you, dear reviewer. We have accepted the comments and corrected in the revised manuscript. |
| 21 | Third sentence, change two-thirds of them to two-thirds of the respondents | Thank you, dear reviewer. Comment accepted and corrected in the revised manuscript. |
| 22 | **Delete the full stop after “**Most (97.2%) of the study participants were currently married” | Thank you, dear reviewer. The full stop has been removed in the revised manuscript. |
| 23 | Kindly delete “(95 % CI; 52.6, 60.6)” from the section women’s comprehensive knowledge of ANC. Do the same for attitude towards ANC uptake. | Thank you, dear reviewer. Comment accepted and corrected in the revised manuscript. |
| 24 | **Conclusion of the study**  **I don’t understand this statement “**The results of this study are looking for relevant stakeholders and health policymakers who pay particular attention to women in rural areas, including advocation regarding maternal health services through mass media” The results are looking for who?? Please delete or rewrite | Thank you, dear reviewer. Revision has been made in the revised manuscript. |
| 25 | There are grammatical errors in the work. Kindly edit the work. | Thank you, dear reviewer. We tried to look for grammatical errors and corrected based on your kind suggestion. |
| 26 | Kindly rewrite this “Moreover, researchers should address the rural women, particularly those who gave childbirth at home and the reason for not using maternal health services should better be addressed through a qualitative study” How can researchers address | Thank you, dear reviewer. The sentence has been re-written in the revised manuscript.  Thank you for the time and commitment you devoted for the improvement of the manuscript. |
