# Supplementary material for: Factors associated with comprehensive knowledge of antenatal care and attitude towards its uptake among women delivered at home in rural Sehala Seyemit district, northern Ethiopia: A community-based cross-sectional study
Source: PLoS One. 2022 Oct 12;17(10):e0276125. doi: 10.1371/journal.pone.0276125 (PMC9555639; doi:10.1371/journal.pone.0276125)
Supplement: Attachment — Submitted filename: Comments.docx [file pone.0276125.s008.docx]

***Data Processing and analysis***

I am still not clear… why didn’t you stick to one (thus, 0.25 throughout). Kindly provide reasons why you used a p-value less than 0.25 at the bivariate level and a p-value of less than 0.05 at the multivariate level. It is still not clear. I am tempted to believe that you used 0.25 to get more values to be significant in the bivariate section for the multivariate results. This to me is a force attempt.

**Conclusion**

This sentence should read *“Stakeholders and health policymakers need to pay particular attention to women in rural areas, including advocation regarding maternal health services through mass media”.*

*You need to edit the work very well.* There are grammatical errors in the work. Kindly edit the work.
