# Supplementary material for: Factors associated with comprehensive knowledge of antenatal care and attitude towards its uptake among women delivered at home in rural Sehala Seyemit district, northern Ethiopia: A community-based cross-sectional study
Source: PLoS One. 2022 Oct 12;17(10):e0276125. doi: 10.1371/journal.pone.0276125 (PMC9555639; doi:10.1371/journal.pone.0276125)
Supplement: Attachment — Submitted filename: Response to Reviewers (R3).docx [file pone.0276125.s009.docx]

July 28, 2022

Manuscript ID: PONE-D-21-01426R2

**Title**: Factors associated with comprehensive knowledge of antenatal care and attitude towards its uptake among women delivered at home in rural Sehala Seyemit district, northern Ethiopia: a community-based cross-sectional study

Journal: PLOS ONE

We would like to thank for Editorial team and reviewers for your constructive comments and valuable improvements to our paper. We appreciate the time and effort that you dedicated to providing feedback on the manuscript. The authors are very happy to re-submit the manuscript. Suggestions and comments have been incorporated and seen with tracked changes in the revised manuscript. Please kindly see a point-by-point response below.

**Response to Reviewer comments**

Reviewer #2 (Dr. Martin Wiredu Agyekum)

Data Processing and analysis

1. I am still not clear… why didn’t you stick to one (thus, 0.25 throughout). Kindly provide reasons why you used a p-value less than 0.25 at the bivariate level and a p-value of less than 0.05 at the multivariate level. It is still not clear. I am tempted to believe that you used 0.25 to get more values to be significant in the bivariate section for the multivariate results. This to me is a force attempt.

**Authors’ response:** Thank you for your insight, the authors taken in to consideration. Much controversy exists regarding proper methods for the selection of variables in confounder control and a number of conclusions reached. Many authors are supposed to use a Wald test for regression to select those variables with a p-value <0.25 in the bivariate logistic regression models for the multivariable regression model.

The problem of the over-selection of the crude analysis by the preliminary significance tests could be overcome by increasing the significance level of the tests. In the rule of using the collapsibility, the test required a level of significance of at least 0.25 to effectively eliminate bias, inflation of test size, and excessive miss rates of the confidence interval, whereas the other rules required a level of significance of at least 0.20. The problems did not arise when the underlying population structure was collapsible, and the choice of the level of significance in these instances did not have a major impact on the inferences about the effect of exposure on disease.

A P value of smaller than 0.25 and other variables of known clinical relevance can be included for further multivariable analysis. A cut-off value of 0.25 is supported by the literature. Therefore, we base this on the Wald test from logistic regression and a p-value cut-off point of 0.25.

Conclusion

2. This sentence should read “Stakeholders and health policymakers need to pay particular attention to women in rural areas, including advocation regarding maternal health services through mass media”.

**Authors’ response:** Thank you for pointing out this, it is now corrected accordingly as follows; ‘The policymakers, program managers, and healthcare providers play a role in awareness creation in the communities for policies prioritizing those disadvantaged groups, have access to the health care which all mothers deserve.’

3. You need to edit the work very well. There are grammatical errors in the work. Kindly edit the work.

**Authors’ response:** Comment accepted and corrected in the revised manuscript
